# SynGAP splice variants display heterogeneous spatio-temporal expression and subcellular distribution in the developing mammalian brain

**DOI:** 10.1111/jnc.14988

**Published:** 2020-03-10

**Authors:** Gemma Gou, Adriana Roca-Fernandez, Murat Kilinc, Elena Serrano, Rita Reig-Viader, Yoichi Araki, Richard L. Huganir, Cristian de Quintana-Schmidt, Gavin Rumbaugh, Àlex Bayés

**Affiliations:** 1 Molecular Physiology of the Synapse Laboratory, Biomedical Research Institute Sant Pau (IIB Sant Pau), Barcelona, Spain; 2 Universitat Autònoma de Barcelona, Bellaterra (Cerdanyola del Vallès), Spain; 3 Nuffield Department of Clinical Neuroscience, University of Oxford, Oxford, UK; 4 Department of Neuroscience, The Scripps Research Institute, Jupiter, FL, USA; 5 Biobank, Biomedical Research Institute Sant Pau (IIB Sant Pau), Barcelona, Spain; 6 Solomon H. Snyder Department of Neuroscience, Johns Hopkins University School of Medicine, Baltimore, MD, USA; 7 Kavli Neuroscience Discovery Institute, Johns Hopkins University, Baltimore, MD, USA; 8 Department of Neurosurgery, Hospital de la Santa Creu i Sant Pau, Barcelona, Spain

**Keywords:** pleiotropy, postnatal development, protein expression pattern, protein isoforms, subcellular localization, SynGAP

## Abstract

The SynGAP protein is a major regulator of synapse biology and neural circuit function. Genetic variants linked to epilepsy and intellectual disability disrupt synaptic function and neural excitability. SynGAP has been involved in multiple signaling pathways and can regulate small GTPases with very different roles. Yet, the molecular bases behind this pleiotropy are poorly understood. We hypothesize that different SynGAP isoforms will mediate different sets of functions and that deciphering their spatio-temporal expression and subcellular localization will accelerate understanding their multiple functions. Using isoform-specific antibodies recognizing SynGAP in mouse and human samples we found distinctive developmental expression patterns for all SynGAP isoforms in five mouse brain areas. Particularly noticeable was the delayed expression of SynGAP-α1 isoforms, which directly bind to postsynaptic density-95, in cortex and hippocampus during the first 2 weeks of postnatal development. Suggesting that during this period other isoforms would have a more prominent role. Furthermore, we observed subcellular localization differences between isoforms, particularly throughout postnatal development. Consistent with previous reports, SynGAP was enriched in the postsynaptic density in the mature forebrain. However, SynGAP was predominantly found in non-synaptic locations in a period of early postnatal development highly sensitive to SynGAP levels. While, α1 isoforms were always found enriched in the postsynaptic density, α2 isoforms changed from a non-synaptic to a mostly postsynaptic density localization with age and β isoforms were always found enriched in non-synaptic locations. The differential expression and subcellular distribution of SynGAP isoforms may contribute to isoform-specific regulation of small GTPases, explaining SynGAP pleiotropy.

## Graphical Abstract

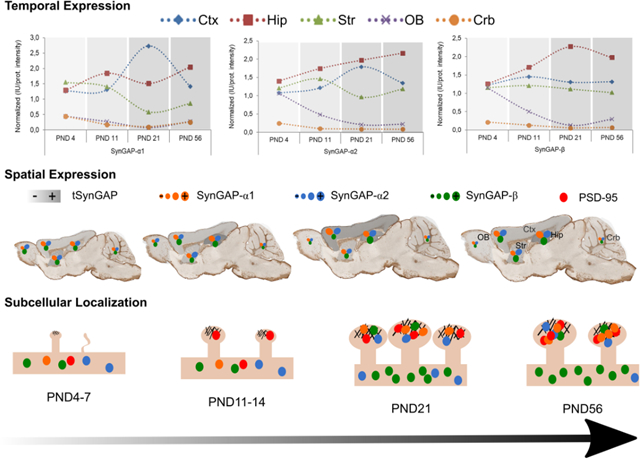

## 1 | INTRODUCTION

De novo mutations in the human *SYNGAP1* gene resulting in genetic haploinsufficiency cause mental retardation type 5 (MRD-5; OMIM #612621), an autosomal dominant form of intellectual disability (ID) with high rates of progressively worsening childhood epilepsy (Agarwal, Johnston, & Stafstrom, 2019; Hamdan et al., 2009; Mignot et al., 2016; Parker et al., 2015; Vlaskamp et al., 2019). This debilitating neurodevelopmental disorder is estimated to be responsible for up to 1% of all cases of ID (Berryer et al., 2013). Studies in mouse models of this condition indicate that a *Syngap1* genetic deficit during specific developmental stages causes premature synaptic maturation in excitatory neurons that result in enhanced neuronal excitability (Aceti et al., 2014; Clement et al., 2012; Clement, Ozkan, Aceti, Miller, & Rumbaugh, 2013; Ozkan et al., 2014). In addition, more recent studies have identified non-developmental functions of the *Syngap1* gene that contribute to memory expression and seizure threshold (Creson et al., 2019). Together, these findings indicate that *Syngap1* is critical for brain cell function. Thus, in depth study of this gene will provide insights into the molecular and cellular processes that contribute to neurological and psychiatric disorders.

*Syngap1* encodes the synaptic Ras/Rap GTPase-activating protein (SynGAP), which was first described as one of the most abundant components of the postsynaptic density (Chen, Rojas-Soto, Oguni, & Kennedy, 1998; Kim, Liao, Lau, & Huganir, 1998). Indeed, this protein regulates the structure and function of excitatory synapses in the mammalian forebrain (Jeyabalan & Clement, 2016; Kilinc et al., 2018). SynGAP has a prominent role in the molecular mechanisms governing synaptic plasticity, being involved in the two hallmarks of this process, incorporation of AMPA receptors into the synaptic plasma membrane (Kim, Lee, Takamiya, & Huganir, 2003; Rumbaugh, Adams, Kim, & Huganir, 2006) and dendritic spine enlargement (Aceti et al., 2014; Vazquez, Chen, Sokolova, Knuesel, & Kennedy, 2004). The activity of SynGAP toward small GTPases is considered to be its key functional role, with the other domains and sequence motifs being involved in regulating it. For instance, the C2 domain is key in the GAP activity toward Rap GTPases (Pena et al., 2008) and phosphorylation determines substrate specificity, as CaMK2α promotes RapGAP activity while CDK5 and PLK2 stimulate RasGAP activity (Walkup, Sweredoski, Graham, Hess, & Kennedy, 2018; Walkup et al., 2015). The exact role of sequences such as the pleckstrin homology (PH) domain, the SH3-binding, or poly-histidine motifs in the function of SynGAP are not yet understood. In vitro studies with purified proteins have shown that SynGAP directly modulates the activity of HRas (Kim et al., 1998), Rap1 (Krapivinsky, Medina, Krapivinsky, Gapon, & Clapham, 2004), Rap2 (Walkup et al., 2015), and Rab5 (Tomoda, 2004). Furthermore, *Syngap1*^±^ mice present increased levels of GTP-bound Rac1 in forebrain extracts (Carlisle, Manzerra, Marcora, & Kennedy, 2008), indicating that SynGAP also regulates Rac1, either directly or indirectly. The GAP activity of SynGAP participates in the regulation of several important signaling pathways for synaptic physiology, such as Ras-MAPK (Komiyama et al., 2002), Ras-PI3K (Qin et al., 2005), Rap-p38 (Krapivinsky et al., 2004; Zhu, Qin, Zhao, Aelst, & Malinow, 2002), and Rac1-PAK (Carlisle et al., 2008).

It remains unclear how SynGAP can have such a broad impact on neuronal signaling. Alternative splicing of *Syngap1* mRNA, which results in many protein isoforms, is likely one mechanism. In mammals, the *Syngap1* gene encodes different protein isoforms that differ in their N- and C-terminus (Chen et al., 1998; Kim et al., 1998; Li et al., 2001; McMahon et al., 2012). The central part of the protein is thus common to all isoforms and accounts for most of it, extending 1,091 residues (>80% of the longest protein isoform) in rat and human. This core region presents a truncated PH domain, lacking the first 24 residues, a C2 domain, a GTPase-activating protein (GAP) domain, a large disordered region of around 600 residues and, finally, a truncated coiled-coil domain lacking its final 11 residues, which is involved in SynGAP multimerization (Zeng et al., 2016). Five N-terminal (A1, A2, B, C, and D) and four C-terminal (α1, α2, β, and γ) SynGAP variants have been described. Of the 20 possible combinations of N- and C-termini with the core region, 13 have been reported either in NCBI, ENSEMBL, or the literature (Chen et al., 1998; Kim et al., 1998; Li et al., 2001). In mouse, SynGAP isoforms will vary in their molecular weight, ranging between 148.3 kDa (SynGAP/A2-α2, the largest) and 121.4 kDa (SynGAP/C-β, the smallest). Isoforms with A1/2, B, and D N-termini present an entire PH domain, while isoforms containing the C N-terminal do not include its first 24 residues. At the other end of the protein, isoforms with C-terminal variants α1, α2, and γ present an entire coiled-coil domain, while those with the β variant lack its last 11 residues. In support of the idea that *Syngap1* alternative splicing alters protein function, the distinct C-terminal spliced sequences have been shown to cause opposing effects on synaptic strength, with α1 driving synaptic depression (McMahon et al., 2012; Rumbaugh et al., 2006) and α2 driving synaptic potentiation (McMahon et al., 2012).

Thus, the multitude of available N- and C-termini likely bestows distinctive functional properties to SynGAP isoforms. However, the expression pattern and subcellular localization of distinct SynGAP isoforms remain largely unexplored, particularly during early postnatal development, when SynGAP is known to have a strong impact on synaptic (Clement et al., 2012, 2013) or dendritic (Aceti et al., 2014; Michaelson et al., 2018) maturation and neuronal development (Muhia, Yee, Feldon, Markopoulos, & Knuesel, 2010; Agarwal et al., 2019). Here, we present a systematic study of the expression of SynGAP isoforms in five different brain regions and four postnatal developmental stages, identifying specific expression patterns for all isoforms, both between brain regions and throughout development. Furthermore, we investigate the differential subcellular localization of SynGAP isoforms and describe how this varies during cortical development. Together, our data illustrates the complexity of SynGAP roles within brain cells, and the key role that C-term variants are likely to play in SynGAP biology. We also find that SynGAP C-termini are important for its subcellular localization and that SynGAP, generally regarded as almost exclusively found at the synapse, is very abundant in the cytosol, specially early in postnatal development, when the brain is most sensitive to *Syngap1* haploinsufficiency (Aceti et al., 2014; Clement et al., 2012; Ozkan et al., 2014).

## 2 | MATERIALS AND METHODS

### 2.1 | Ethics statement and procedures on human cortical samples

All surgical procedures were approved by the Ethics Committee on Clinical Research from the Hospital de la Santa Creu i Sant Pau (approval reference number 16/041). All samples collected originated from neuro-oncological surgery unit at the Hospital de Sant Pau i la Santa Creu between 2016 and 2018. Adult healthy cortical samples, as determined by pre-surgery nuclear magnetic resonance, were collected in those cases that a corticectomy had to be performed to access subcortical pathological tissue. All patients were informed and signed an informed consent. Resected tissue was rapidly wrapped in aluminum foil and snap-frozen in liquid nitrogen. Samples were stored in −80°C.

### 2.2 | Ethics statement on animal research and animal handling

**FIGURE 1:**
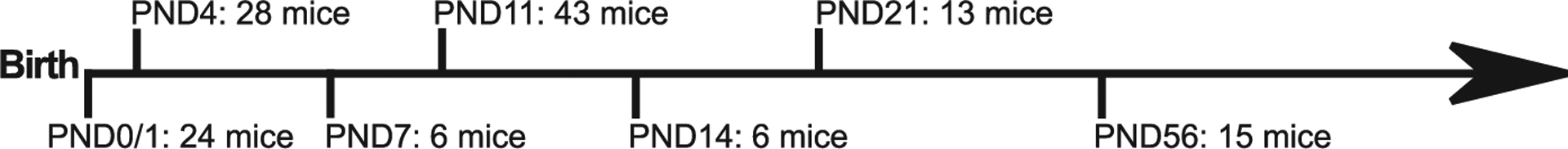
Study timeline for developmental expression and subcellular localization studies. Postnatal day (PND) indicates postnatal day of tissue collection. The total number of animals used for discovery MS-based studies, spatio-temporal expression and subcellular distribution of SynGAP and its isoforms at each PND is also indicated

All procedures, unless specifically indicated, were done with C56BL/6J mice (Jackson Laboratories, Research Resource Identifier, RRID:MGI:5656552) in accordance with national and European legislation (Decret 214/1997 and RD 53/2013). These were approved by the Ethics Committee on Animal Research from the Institut de Recerca de I’Hospital de la Santa Creu i Sant Pau (IR-HSCP) and the Departament de Territori i Sostenibilitat from the Generalitat de Catalunya (approval reference num. 9,655). Maintenance, treatment and experimental procedures with mice were conducted at the Animal Facility of the IR-HSCP. Mice were housed at a 12 hr light/dark cycle with fresh water and food ad libitum. No more than five mice of a given gender were place in the same cage. Special chow (T.2019.12, Envigo) was administered to pregnant mothers and litter until weaning (postnatal day [PND] 21), whereas adult mice were fed with regular chow (T.2014.12; Envigo). The total number of animals used to conduct mass spectrometry (MS)-based, spatio-temporal expression, and subcellular localization studies was 135. The specific number of animals used per age was: PND0/1:24, PND4: 28, PND7: 6, PND11: 43, PND14: 6, PND21: 13, PND56: 15 (See Figure 1). For ages between PND0 and 21, female and male mice were used at equal ratios. PND56 mice were males. Mice culling between PND0 and 4 was performed by head dissection or by cervical dislocation from that age onwards without the use of anesthetic in any case and minimizing animal suffering. All experiments were conducted from 9 a.m. to 9 p.m. and those animals that were clearly smaller than the rest of the littermates were excluded for subsequent analyses.

### 2.3 | Mouse brain dissection

Mouse heads were soaked with chilled 1× phosphate-buffered saline (PBS, 0.144 M NaCl, 2.683 KCl mM, 10.144 mM Na2HPO4, 0.735 mM KH2PO4, [P5368-10PAK from Sigma]) and dissected using scalpel blades while placed onto a glass petri dish with a filter paper (Merck-Millipore). The skull and meninges were removed from brain using Iris scissors (PMD120; Thermo Scientific) and tissue forceps 1:2 (PMD023445; Thermo Scientific). For brain dissection of PND0-7 animals a magnifying loupe (Olympus KC 1,500 Ledplus; Olympus) was used. Brain areas were dissected as previously described (Spijker, 2011). Tissue weight was recorded before snap-freezing in liquid nitrogen and stored in a −80°C freezer.

### 2.4 | Anti-SynGAP-β antibody generation

SynGAP-β antibody was raised against SynGAP aa.1273–1285 at the research laboratory of Prof. Richard L. Huganir, Johns Hopkins University. The antigen peptide with N-terminus Cysteine (NH2-CGGGGAAPGPPRHG-COOH) was coupled with keyhole limpet hemocyanin (77,600; Thermo Fisher). The antigen was injected into rabbit and antisera were collected after primary and several booster injections. Antisera were further purified with affinity column containing sulfo-link coupling resin (20,401; Thermo Fisher) coupled with same antigen peptide. This antibody will be shared upon reasonable request.

### 2.5 | Total protein extraction, subcellular fractionation and protein quantification

For extraction of total proteins, samples were mixed with chilled buffer (50 mM Tris-HCl pH 9 [T1503-1KG], 1% sodium deoxycholate [30970-100G], 50 mM NaF [106449, Merck-Millipore], 20 mM ZnCl2 [96468-50G], 1 mM sodium orthovanadate [S6508-10G], 1:2,500 phenyl methane sulfonyl fluoride [P7626-5G], 2 μg/ml aprotinin [616370-10MG, Merck-Millipore], and 2 μg/ml leupeptin [108976-10MG, all from Sigma-Aldrich unless indicated]) at a 1:17.5 tissue:extraction buffer ratio (g/ml). Brain tissue was homogenized by 30 strokes in 1 or 7-ml borosilicate Dounce homogenizers (357542 & 357544, glass-Teflon tissue grinder; Wheaton) depending on the volume of buffer required. Then, it was incubated on ice for 1 hr and centrifuged at 21,000 *g* for 30 min at 4°C in 1.5 ml centrifuge tubes (3810x; Eppendorf). The resulting pellet was re-homogenized twice following the same procedure and resulting supernatants were pooled. In the last re-homogenization cycle half of the initial w/v ratio was used. Prior to protein quantification, 1% sodium dodecyl sulfate (SDS) (428029-1EA; Merck-Millipore) was added to all samples.

Subcellular fractions were prepared following previously described procedures (Bayés et al., 2012; Carlin, Grab, Cohen, & Siekevitz, 1980). All centrifugation steps were done at 4°C and samples were always kept in ice. Briefly, tissue was homogenized using 7-ml glass-Teflon tissue grinders (357,544, borosilicate Dounce homogenizer; Wheaton). A 1:9 ratio was used and ~40 strokes were applied. Next, a 10 min centrifugation (Epp 5417R; Eppendorf) at 1,400 *g* was conducted. The resulting supernatant was conserved and the pellet was subjected to two re-homogenizations in the same conditions. The three pooled supernatants were centrifuged at 700 *g* for 10 min, this sample corresponds with the S1 fraction. This was centrifuged 30 min at 21,000 *g*. The resulting soluble fraction was considered the cytosolic fraction, whereas the pellet obtained contained all membranes. This was resuspended with sucrose 0.32 M and 50 mM Tris pH 7.4. A sucrose gradient was prepared with 1 ml of (top to bottom): sample, 0.85 M sucrose and Tris 50 mM pH 7.4; 1 M sucrose and Tris 50 mM pH 7.4, and 1.2 M sucrose and Tris 50 mM pH 7.4. Then, this gradient was centrifuged with a SW60 Ti rotor (Beckman Coulter) at 82,500 *g* for 2 hr. The interphase between sucrose 1 and 1.2 M was recovered to obtain the synaptosome fraction. The rest of the gradient was centrifuged at 50,000 *g* 30 min in a fixed rotor and the resulting pellet, containing the non-synaptic membrane (NSM) fraction, was resuspended with 1% SDS and 50 mM Tris pH 7,4. The synaptosome fraction was diluted to reach a final concentration of 10% sucrose with Tris 50 mM pH 7.4 and centrifuged in an Epp 5117R centrifuge (Eppendorf) at 21,000 *g* during 30 min using 1.5 ml tubes. The resulting pellet was resuspended in Tris 50 mM pH 7.4, 1% Triton X-100 (93443-100Ml; Sigma-Aldrich) and maintained in ice for 10 min. Finally, samples were centrifuged at 21,000 *g* during 30 min. As a result, Triton X-100 soluble fraction, referred as synaptic non-PSD (SNP), and the Triton X-100 insoluble fraction enriched in postsynaptic densities (PSDs), were obtained. Fraction protein yield was defined as the ratio of total protein amount (μg) by tissue weight (mg).

Protein concentration was determined using a micro-BCA protein assay kit (10249133; Thermo-Fisher Scientific). Prior to IB protein concentrations were corrected by silver stain (1610449, Silver Stain Plus^™^ kit; Bio-Rad).

### 2.6 | Protein dialyzation for detergent exchange

Dialysis was used to exchange sodium deoxycholate with Triton X-100 from total protein extracts prior to immunoprecipitation (IP). Membranes for dialysis (Visking Corporation) were activated according to manufacturer’s instructions. Samples were dialyzed over-night (ON) against the dialysis buffer (50 mM Tris HCl pH7.4 and 1% Triton X-100) at a v/v ratio of 1:1,000 in constant agitation at 4°C. After dialysis, Triton X-100 concentration was adjusted to 1% if required. Finally, samples were sonicated with an ultrasonic bath Sonicator (Thermo Fisher Scientific) at 5% of its maximum intensity during 45 s with 1.45 s on/off cycles.

### 2.7 | Immunoprecipitation

IPs were performed on total protein extracts from cortical samples at different ages. All IPs were performed at a protein concentration of 8 mg/ml. All steps were performed at 4°C in an orbital agitator (Stuart). The following amounts of protein were used for each IP: 9 mg for PND0/1 (*n* of mice = 24), 16 mg for PND11 (*n* of mice = 15), 8 mg for PND21 (*n* of mice = 3), and 7 mg for PND56 (*n* of mice = 3) samples. IPs were performed as described by the kit manufacturer (26,147, Pierce® Direct IP Kit; Thermo Fisher Scientific). A sepharose resin (Sigma P3391-250MG) was washed four times with conditioning buffer (50 mM Tris pH 7.4). Next a sample pre-clearing step was performed by mixing it with washed resin for 2 hr at 4°C. Pre-cleared sample was mixed with an anti-SynGAP antibody recognizing an epitope common to all its isoforms (5540S; Cell Signaling Technology [RRID:AB_10695900]) at a 1:15 (v:v) ratio ON. Each 200 μL of pre-cleared sample were incubated with 7.5 μL of A sepharose resin during 3 hr. A 100 *g* centrifugation step in a column was performed to recover the resin. Resin was washed three times with dialysis buffer and once with conditioning buffer. Bound protein was eluted with 15 μL of the acidic elution buffer from the kit during 10 min.

### 2.8 | Protein electrophoresis

Protein samples for electrophoresis were prepared with 1× Laemmli loading sample buffer (50 mM Tris-HCl, pH 6.8; 2% SDS; 1% β-mercaptoethanol [M6250-100 ml], and 0.04% bromophenol blue [B5525-5g, all from Sigma-Aldrich]) and 10% glycerol (Sigma-Aldrich) and heated at 95°C for 5 min. TGX Stain-Free^™^ gels (161-0181 & 161-0185, SF gels; Bio-Rad) were prepared and activated according to manufacturer’s instructions. All blue or kaleidoscope precision plus protein standards (Bio-Rad) were used as well as a vertical MiniProtean system kit (Bio-rad) and 1× running buffer (0.025 M TRIS pH 8.4; 0.187 M glycine [G8898-1KG; Sigma-Aldrich] and 0.1% SDS). Electrophoretic conditions were 25 mAmp per each 0.75 mm wide gel or 50 mAmp per each 1.5 mm wide gel.

Proteins resolved in SDS–PAGE gels were stained ON at 22°C with *Coomassie* solution (B8522-1EA; Sigma-Aldrich). Gels were washed with 2.5% acetic acid (45740-1L-F; Sigma-Aldrich) and 20% methanol during 10 min in a rocking platform shaker (Stuart) and later with subsequent washes of 20% methanol, until protein bands were clearly visible. Gel images were acquired with ChemiDoc XRS+ (Bio-Rad) and quantified with Image Studio Lite ver. 3.1 (LI-COR Biosciences).

### 2.9 | Immunoblot

Protein transference was conducted using the MiniProtean kit (Bio-Rad), and 1× chilled transference buffer (20% methanol [A3493.5000; Panreac]; 39 mM Glycine; 48 mM TRIS; 0.04% SDS). Proteins were transferred into methanol pre-activated polyvinylidene fluoride (PVDF) membranes (IPFL00010, Immobilon-P; Merck-Millipore). After transference, PVDF membranes were blocked with 5 ml Odissey blocking solution (927-50000; LI-COR) prepared with 1× tris-buffered saline (TBS) [50 mM Tris·HCl pH7.4; 1,5 M NaCl [443824T]) and 0.1% sodic azide [S2002-100G, all from Sigma-Aldrich]) and incubated in a roller mixer (Stuart) with primary antibody solution ON at 4°C or 2 hr at 22°C. Commercial primary antibodies were: total SynGAP (tSynGAP) (NBP2-27541; Novus Biologicals, [RRID:AB_2810282] and Thermo PA1-046, [RRID:AB_2287112], only in Figure S2) at 1:2,500 and 1:2,000 dilution, SynGAP-α1 (06-900; EMD Millipore, [RRID:AB 1163503]) at 1:1,000, SynGAP-α2 (04-1071 [EPR2883Y]; Merck-Millipore, [RRID:AB_1977520]) used at 1:2,000 dilution, PSD-95 (3,450; Cell Signaling, [RRID:AB_2292883]) at 1:1,000, Gephyrin (ab32206; Abcam, [RRID:AB_2112628]) at 1:500, CaMK2α (05-532; Merck-Millipore, [AB_309787]) at 1:1,000, GAD67 (MAB5406 [1G10.2]; Merck-Millipore, [RRID:AB_2278725]) at 1:500, Synaptophysin (Ab8049; Abcam [SY38], [RRID:AB_2198854]) at 1:1,000 and GAPDH (ab9484; Abcam, [RRID:AB_307274]) at 1:500 dilution. Membranes were washed four times with 1× T-TBS for 5 min before incubation for 1 hr at 22°C protected from light with 5 ml of the following secondary antibodies. Secondary antibodies were prepared with T-TBS (50 mM Tris·HCl pH7.4, 1.5 M NaCl, 0.1% Tween20, all from Sigma-Aldrich) at 1:7,500 dilution: anti-rabbit (926-68073, IRDye 680CW, [AB_10954442]), anti-mouse (926-32212, IRDye 800CW [RRID:AB_621847] or 925-68072, IRDye 680RD, [RRID:AB_2814912]) and anti-goat (926-32214, IRDye 800CW, [RRID:AB_621846]). Membranes were re-blotted without prior stripping by an ON incubation at 4°C or 2 hr at 22°C, depending on the antibody. Images were acquired with an Odissey Scanner (LI-COR Biosciences) and protein bands were analyzed with Image Studio Lite ver. 3.1 software (LI-COR Biosciences). Membranes transferred from TGX Stain-Free^™^ gels were imaged and quantified for posterior normalization steps prior to blocking with a ChemiDoc XRS+ (Bio-Rad) using the Image Lab software (Bio-Rad).

### 2.10 | Normalization of immunoblot data

In spatio-temporal protein expression studies, band intensity units (IUs) were first corrected for immunoblot technical variability using the value of total protein transferred to PVDF membranes obtained from the TGX Stain-Free^™^ quantification (IU/protein intensity). Corrected IUs were then normalized using the average IU of all bands in a blot. This normalization removed the technical variability between blots allowing to accumulate data from immunoblot replicates.

In subcellular localization studies, we first corrected band IU (e.g. tSynGAP in PSD) per amount of total protein used for immunoblotting (e.g. tSynGAP IU in PSD/μg PSD protein). These values were next multiplied by protein yield (with units: μg protein/mg tissue) of their corresponding subcellular fraction, which retrieved a value of specific protein abundance per fraction (e.g. tSynGAP IU in PSD/mg tissue). Finally, these values were normalized by the abundance in the starting homogenate (S1 fraction; e.g. tSynGAP IU in PSD/tSynGAP IU in S1). This normalization step allowed accumulating data from immunoblot replicas and compare subcellular distribution between antibodies.

### 2.11 | Sample preparation and mass spectrometry-based proteomics

Proteins were separated by SDS-PAGE and were stained with *Coomassie* (Bio-Rad). Bands between ~120–200 kDa were excised from acrylamide gels in a transilluminator (22V; Cultex) using scalpel blades. Excised gel bands were subjected to an in-gel digestion protocol being first reduced with 10 mM dithiothreitol (Sigma-Aldrich) and alkylated with 55 mM iodoacetamide (8.04744.0025; Sigma-Aldrich), and later digested with trypsin (V5111; Promega Biotech Ibérica). Tryptic peptides were eluted from acrylamide and around 80% of each trypsin-digested sample was injected in a linear trap quadrupole (LTQ) Orbitrap VelosPro with a short chromatographic method (40 min gradient) in a 25 cm 1.9 μm column. To avoid carry over, BSA runs were added between samples. BSA controls were included both in the digestion and LC-MS/MS analyses for quality control. This experiment was done twice. The data were searched using an internal version of the search algorithm Mascot (Matrix Science) against a SynGAP (May 2014) homemade database. The Mascot database server search was done with Protein Discoverer ver. 1.4.1.14 (DBVer.:79) using the following search parameters: mass precision of 2 ppm; precursor mass range of 250 Da to 5,000 Da; Trypsin with a maximum of three miss-cleavages; the peptide cut-off score was set at 10 and peptide without protein cut-off was set at 5. Peptides were filtered based on IonScore >20. The precursor mass tolerance (MS) was set at 7 ppm and fragment mass tolerance (MS/MS) was set at 0.5 Da with two variable modifications: oxidation (M) and acetylation (protein N-term), and one fixed modification (C): carbamidomethyl. False discovery rates determined by reverse database searches and empirical analyses of the distributions of mass deviation and Mascot Ion Scores were used to establish score and mass accuracy filters. Application of these filters to this dataset was below 1% false discovery rates as assessed by reverse database searching.

### 2.12 | Primary neuronal and cell culture, transfection, fluorescent immunostaining, and imaging

Hippocampal neurons from PND0 *Syngap1*^*flox*/flox^ (Clement et al., 2012, 2013) mice were plated on poly-D-lysine (P6407; Sigma-Aldrich) coated coverslips and infected with AAV.CaMK2α. Cre (RRID:Addgene_105558) to obtain *Syngap1*^−/−^cells. Cultures were maintained in Neurobasal-A media (12348–017) containing 10 μg/ml Gentamycin (15750060), 2 mM Glutamax (35050061), and 2% B27 (17504044, all from GIBCO). At days in vitro (DIV) 4, cells were treated with 1 μM Ara-C to prevent excessive glial proliferation. At DIV18, cells were transfected with plasmids encoding enhanced green fluorescent protein-tagged full-length SynGAP C-terminal isoforms using NeuroMag (NM50500, OZBiosciences) in accordance with manufacturer’s instructions. Following an ON incubation, neurons were fixed for 5 min at 22°C in PBS containing 4% paraformaldehyde (PFA)/4% sucrose and thoroughly washed with PBS. Neurons were permeabilized with 0.2% Triton-X for 10 min and blocked with 10% normal goat serum in PBS for 1 hr. Samples were then incubated with Alexa Fluor 488 conjugated anti-enhanced green fluorescent protein antibody (A-21311; Thermo Fisher Scientific, [RRID:AB_2214]) in PBS with 5% goat serum at 4°C ON. Coverslips were washed multiple times with PBS and mounted onto glass slides using Prolong Glass mounting medium. Images were obtained using a FV1000 Olympus laser scanning confocal microscope. For anti-SynGAP-beta antibody validation, HEK293T Cells (Kind gift of Joseph Kissil) were cultured in DMEM media containing 10% fetal bovine serum and penicillin/streptomycin. Pools of these cells were transfected with GFP-tagged SynGAP cDNAs containing one of the four known C-term spliced sequences.

### 2.13 | Data statistical analyses

Statistical tests used are indicated in figure legends, together with the exact number of biological and technical replicates. GraphPAD Prism ver. 6.0 (GraphPad) was used to conduct statistical analyses. When required data were assessed for normal distribution by descriptive statistic measures (mean and median) and applying the Shapiro-Wilk and Kolmogorov–Smirnov tests. All statistical analyses were conducted with a significance level of α = 0.05 (*p* ≤ .05). No test for outliers was done, no data points were excluded and no blinding was performed. No statistical method was used to determine sample size, which was determined based on the previous experience of the group with the goal to minimize the number of animals required. Also, no randomization was performed to allocate subjects in this study and this study was not pre-registered.

## 3 | RESULTS

### 3.1 | Total SynGAP protein expression is different between brain regions and changes throughout postnatal development

**FIGURE 2:**
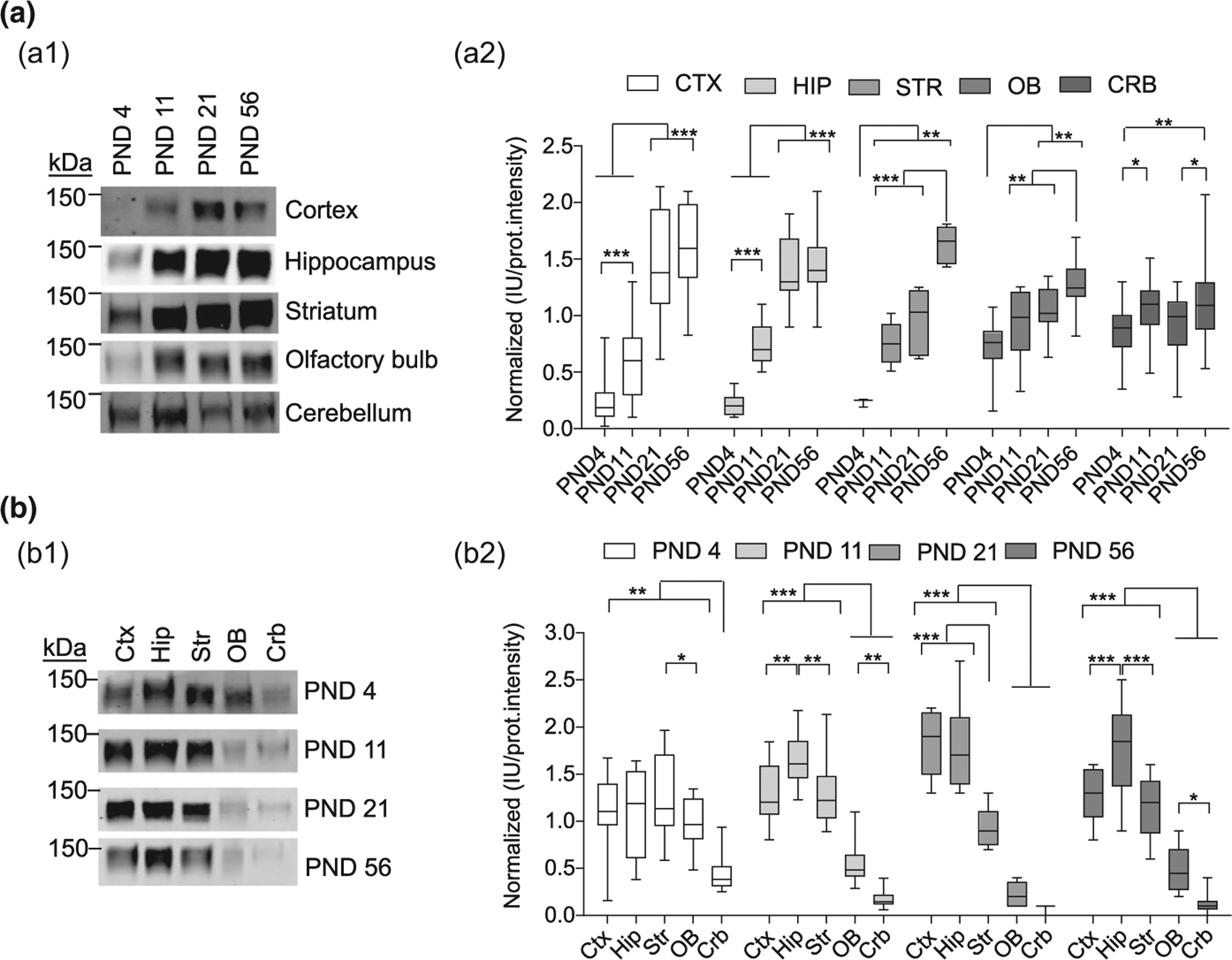
Abundance of total SynGAP (tSynGAP) in five different brain regions and four postnatal stages. (a) Developmental changes in tSynGAP abundance in five different brain regions (cortex, hippocampus, striatum, olfactory bulb, and cerebellum). Ages investigated were postnatal day (PND) 4, 11, 21, and 56. (a1) Representative immunoblots showing tSynGAP abundance in each of the five tissues. (a2) Box and whiskers plots depict the mean of normalized protein abundance data derived from immunoblot intensity (*N*: cortex 14–20, hippocampus 12–16, striatum 6–7, olfactory bulb 11–16, and cerebellum 23–24). *N* indicates total number of technical replicates from a pool of a given brain area coming from of a minimum of two mice. The standard error of the mean (*SEM*) is also shown. Mean differences were analyzed by one-way ANOVA followed by Tukey’s post-hoc test, ***p < .001, **p < .01, and *p < .05. (b) Brain region changes in tSynGAP abundance in four life stages, including three postnatal development stages (PND4, 11, and 21) and adulthood (PND56). (b1) Representative immunoblots showing tSynGAP abundance in each life stage. (b2) box and whiskers plots depict the mean of normalized protein abundance data derived from immunoblot intensities (*N*: cortex 6–15, hippocampus 6–15, striatum 6–15, olfactory bulb 6–15, and cerebellum 6–15). *N* indicates total number of technical replicates from a pool of a given brain area coming from of a minimum of two mice. The standard error of the mean (*SEM*) is also shown. Mean differences were analyzed by one-way ANOVA followed by Tukey’s post-hoc test, ***p < .001, **p < .01 and *p < .05

Using an antibody that recognizes a sequence common to all SynGAP isoforms we have analyzed by immunoblot the abundance of all of them together, what we have called total SynGAP (tSynGAP). We have investigated tSynGAP expression in five mouse brain regions (cortex, hippocampus, striatum, olfactory bulb, and cerebellum) at 4, 11, 21, and 56 postnatal days (PND) of life (Figure 2). Depending on the tissue, tSynGAP presents three patterns of developmental expression (Figure 2a). In cortex and hippocampus, tSynGAP increases sharply, reaching its maximum at PND21, and remaining at this level until PND56. Between PND4 and PND21, tSynGAP levels increase over six times in both tissues. Striatum presents a different pattern: tSynGAP expression is maintained constant between PND11 and PND21, and its maximum level is not reached until PND56. Yet tSynGAP levels also increase notably, also around six times, between PND4 and 56. Finally, both the olfactory bulb (OB) and the cerebellum present a very modest, albeit significant, increase in tSynGAP levels. Between PNDs 4 and 56, tSynGAP increases 1.7 times in OB and 1.2 in cerebellum.

We have also investigated how tSynGAP levels compare between tissues at each of these four developmental stages (Figure 2b). Early in postnatal development tSynGAP levels are very similar in cortex, hippocampus, striatum, and OB, while cerebellum already presents the lowest levels. At PND4 there is approximately 2.5 times more tSynGAP in forebrain regions than in cerebellum. This difference becomes larger with age, reaching a maximum difference of 30 times when comparing hippocampal and cerebellar expression at PND21/56 or cortical and cerebellar expression at PND21. As mice develop, the levels of tSynGAP in OB also lag behind those of the other forebrain areas (Figure 2b), being the maximum difference at PND21, when cortex and hippocampus express seven times more tSynGAP than OB. At PND11, cortex and striatum display similar levels of tSynGAP, while hippocampus presents a slight, but significantly higher abundance, being the tissue with the highest tSynGAP levels at this age. At PND21, cortical and hippocampal tSynGAP have similar levels, presenting almost twice as much tSynGAP than striatum. This is in agreement with the sustained tSynGAP levels previously observed in striatum between PND11 and 21 (Figure 2a). Finally, at PND56, the abundance profile of tSynGAP at cortex, hippocampus, and striatum is very similar to that found at PND11. Cortex and striatum have similar abundance, while hippocampus presents significantly more tSynGAP.

### 3.2 | In silico identification and experimental validation of novel *Syngap1* splice variants

ENSEMBL, NCBI-Gene, and UniProt (as of 02 May 2019), together with the previous literature (Chen et al., 1998; Kim et al., 1998), report a total of 15, 9, and 7 *Syngap1* transcripts in mouse, rat, and human respectively (Table S1). Remarkably, there is still little overlap between these databases. For instance, in mouse, only two proteins can be directly related between ENSEMBL and NCBI. Interestingly, the NCBI Gene database identifies unpublished variants in mice (four N-terminal and one C-terminal). We refer to these unreported N-terminals as A3, A4, E, and F, whereas the C-terminal one as α3 (Table S1 and Figure S1). The first two N-term variants are shorter versions of A1/2, E presents a unique N-terminus, and F starts at residue 430 inside the core of SynGAP. If the F variant is expressed at the protein level, it would lack the PH, C2, and GAP domains, which could be functionally relevant to neuronal biology. In order to investigate if any of these predicted variants is expressed at the protein level, we immunoprecipitated tSynGAP from mouse cortex at four postnatal stages (PND0/1, 11, 21, and 56) and performed high-throughput MS-based proteomics. However, we could not identify unique peptides for any of these variants. Instead, we identified a unique peptide corresponding to the first residues of the D N-terminus (Table S2), which had only been reported at the RNA level (Li et al., 2001). Importantly, this peptide presented an acetylated initial methionine, which is a common post-translational modification of the N-terminus (Varland, Osberg, & Arnesen, 2015). The rat D variant was originally submitted (Li et al., 2001) to NCBI as an artifactual sequence resulting from the fusion of transcripts from two different genes, as reported later (McMahon et al., 2012), and thus considered nonexistent. Yet, our proteomics experiments identify the acetylated N-terminus of the D variant in cortical samples from all ages investigated. The recurrent identification of this acetylated N-terminal peptide provides strong evidence for the expression of this variant in mouse cortex.

### 3.3 | SynGAP isoforms present different developmental expression patterns

**FIGURE 3:**
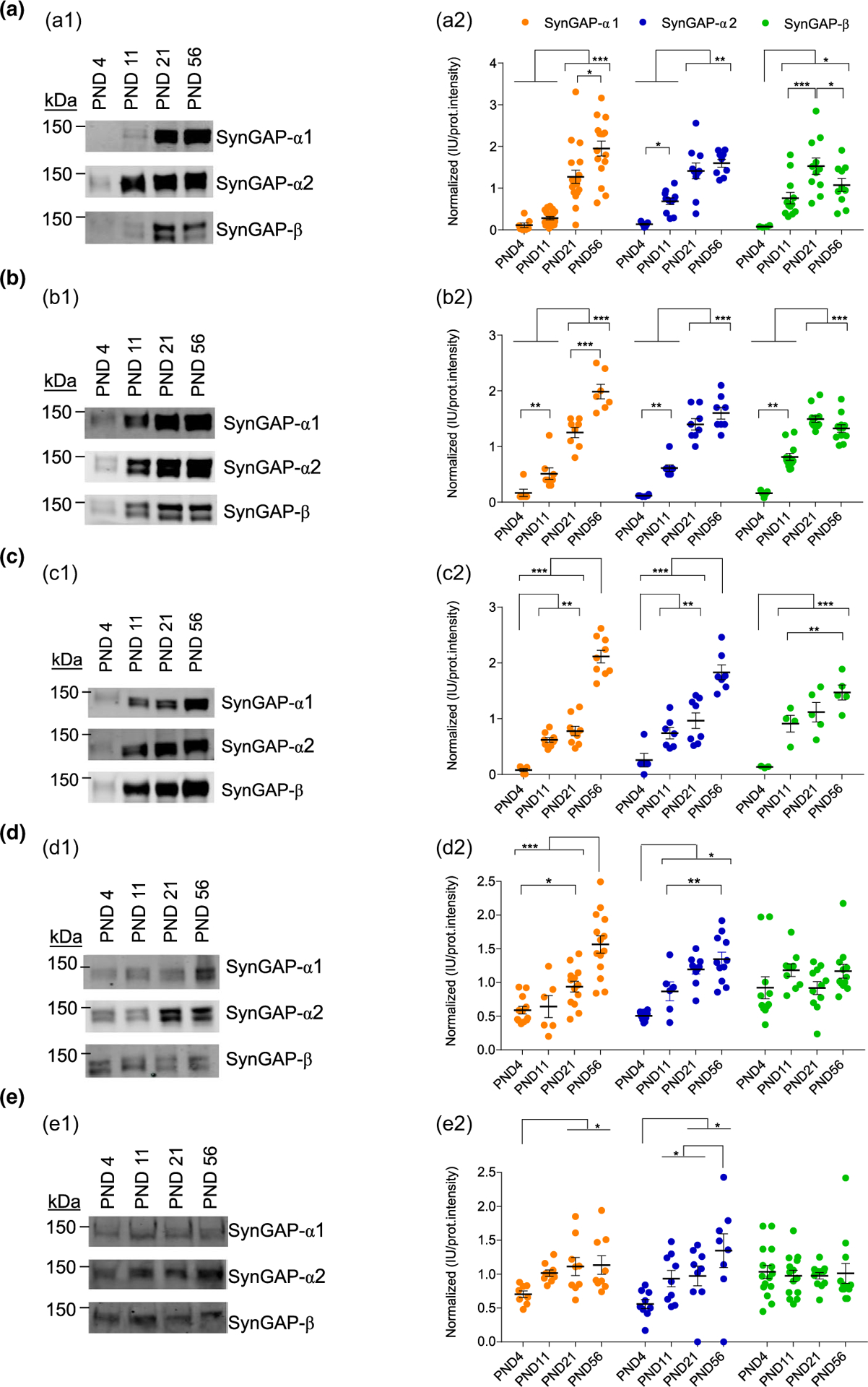
Compared protein abundance of SynGAP isoforms along postnatal development in five different brain regions. (a–e) data from cortex, hippocampus, striatum, olfactory bulb, and cerebellum respectively. (a1–e1) representative immunoblots for SynGAP isoforms containing each of the three C-terminal variants (α1, α2 and β). (a2–e2) dot plots for each brain region depicting mean normalized protein abundance data from each isoform derived from immunoblot intensities (*N*: cortex 4–19, hippocampus 6–12, striatum 3–9, olfactory bulb 6–14, and cerebellum 8–15). *N* indicates total number of technical replicates from a pool of a given brain area coming from a minimum of two mice. The standard error of the mean (*SEM*) is also shown. Mean differences were analyzed by one-way ANOVA followed by Tukey’s post-hoc test, ***p < .001, **p < .01, and *p < .05

**FIGURE 4:**
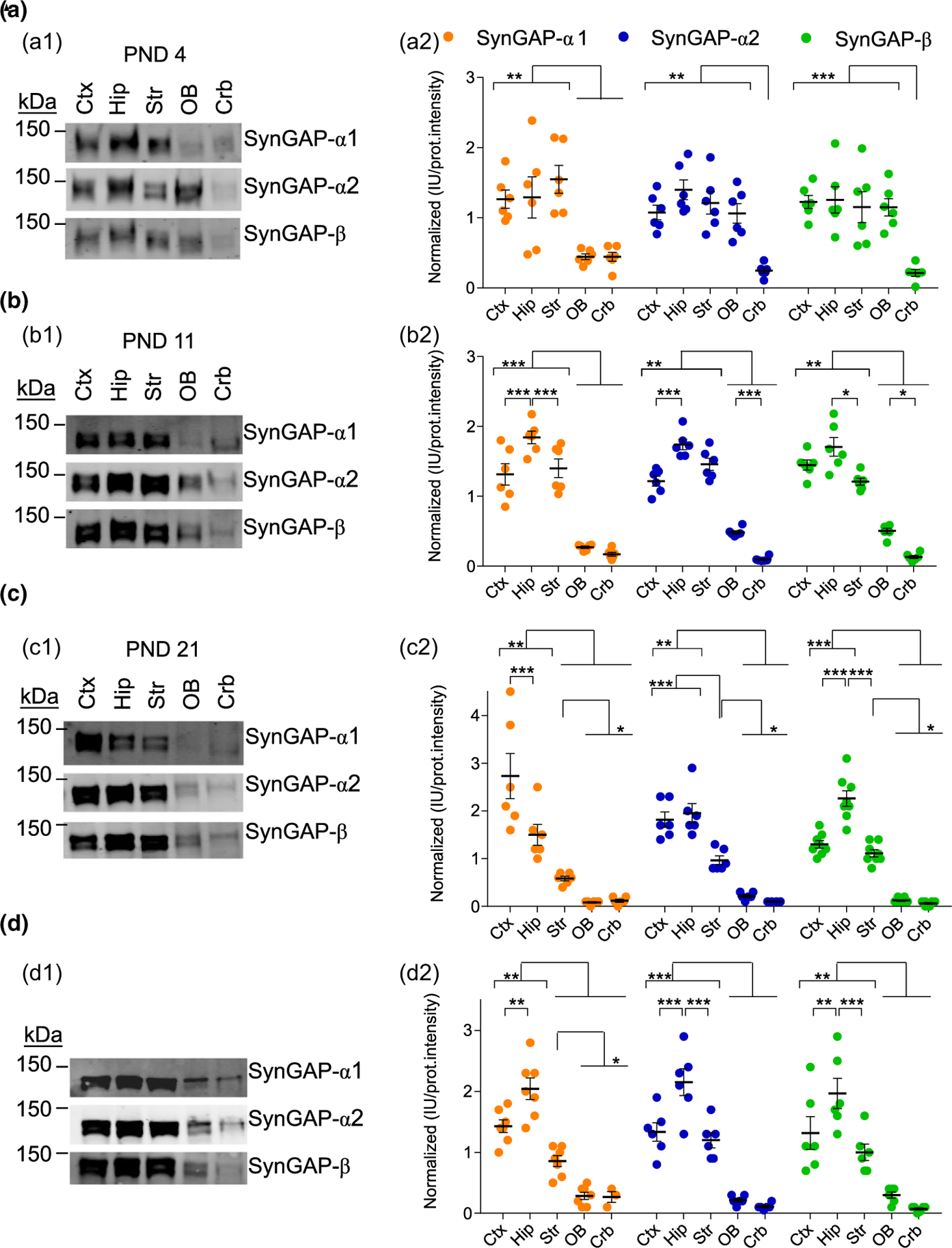
Compared protein abundance of SynGAP isoforms in five different brain areas along postnatal development and in adulthood (a–d) data from postnatal day (PND) 4, 11, 21, and 56, respectively. (a1–d1), representative immunoblots for SynGAP isoforms containing each of the three C-terminal variants (α1, α2, and β). (a2–d2), Dot plots for each life stage depicting normalized protein abundance data from each isoform derived from immunoblot intensities (*N*: cortex 6, hippocampus 6, striatum 6, olfactory bulb 6, and cerebellum 6). *N* indicates total number of technical replicates from a pool of a given brain area coming from of a minimum of two mice. The standard error of the mean (*SEM*) is also shown. Mean differences were analyzed by one-way ANOVA followed by Tukey’s post-hoc test, ***p < .001, **p < .01, and *p < .05.

SynGAP isoforms present four different C-terminal variants that have been identified at the protein level, which are named alpha1 (α1), alpha2 (α2), beta (β), and gamma (γ). Commercial antibodies are available for two (α1 and α2) and we raised a new antibody that recognizes the β sequence. We confirmed that these three antibodies are selective by showing that they do not cross-react (Figure S2). In our experimental conditions, as in previous works (Kim et al., 1998; Li et al., 2001; McMahon et al., 2012; Yang, Tao-Cheng, Bayer, Reese, & Dosemeci, 2013), these C-terminal specific antibodies distinguish two major bands (Figures 3–4). These two bands correspond with at least two different isoforms, which will necessarily present different N-terminus. Notably, we have not found statistically significant abundance differences between the top and bottom bands in any of the experiments performed. This indicates that isoforms with the same C-terminus display equivalent expression patterns along development in the five brain regions investigated. For this reason, we considered both bands together for subsequent analysis.

In cortex (Figure 3a), α1-containing SynGAP isoforms remain at very low levels until PND11 as compared with their maximum expression. Between PND11 and PND21, α1 expression increases fivefold, to reach over 60% of their adult (PND56) levels. In contrast, isoforms containing α2 and β C-term variants already present around 50% of their maximum abundance at PND11. Interestingly, α1-, α2-, and β-containing isoforms vary in their pattern of cortical expression. Namely, α1 isoforms do not reach their maximum until PND56, while α2 and β isoforms peak at PND21. Furthermore, while α2 isoforms maintain their maximum expression level between PND21 and 56, those of β isoforms decrease significantly after PND21, presenting 70% of their maximum expression at PND56.

The hippocampal expression pattern (Figure 3b) of the isoforms investigated is quite similar to that of cortex. α1 isoforms reach their maximum expression at PND56, while α2 does it at PND21 and β isoforms peak at PND21. Here, we also observed a decrease in β isoforms between PND21 and 56, although it did not reach statistical significance. Also, the abundance of α1 isoforms does increase between PND4 and 11, as opposed to what we observed in cortex. Still, α1 isoforms expression fold change between PND11 and PND56 is higher (4-fold) than that observed between PND4 and 11 (2-fold).

In striatum (Figure 3c), α1 and α2 isoforms present a biphasic expression pattern that we have not observed in any other tissue. Expression increases from PND4 to PND11 and then again between PND21 and PN56, but during the second and third weeks (PND11–21) the expression of these isoforms remains constant. Striatal levels of β isoforms suggest a similar pattern, as PND56 expression is higher than PND11 and there is no difference between PND11 and PND21. Yet, the difference between PND21 and PND56 does not reach statistical significance. Thus, our data could also be interpreted as that β isoforms reach their maximum level at PND21 and then this is maintained.

In the OB and cerebellum (Figure 3d,e) we observed less developmental variation in the abundance of SynGAP isoforms, particularly for β isoforms, which do not present any difference in their expression along the postnatal period investigated. In OB, both α1 and α2 isoforms present a moderate increase in their expression level, showing a maximum at PND56. Finally, in cerebellum, α1 levels are constant from PND11 onwards, while α2 present a biphasic increase in expression, with a period of latency between PND11 and PND21.

Overall, we observed a much better correlation in the developmental expression of α1 and α2 isoforms, as this is statistically significant in all brain areas but OB and cerebellum, than between α1 and β isoforms, which were never found significantly correlated (Table 1). β isoforms present a better expression correlation with α2 isoforms, reaching statistical significance in hippocampus, what suggests that α2 isoforms would present an intermediate expression pattern between those of SynGAP α1 and β-containing isoforms.

### 3.4 | Developmental expression pattern of SynGAP-α1/α2 isoforms follows the formation of excitatory and inhibitory synapses

We also sought to explore if the developmental expression profile of SynGAP isoforms could be linked to the temporal acquisition of excitatory and inhibitory synapses and/or neurons. To achieve this purpose we compared the expression of SynGAP isoforms to that of PSD-95, marker of excitatory synapses; Gephyrin, marker of inhibitory synapses; CaMK2α, marker of excitatory neurons; and GAD-67, marker of inhibitory neurons (Table 1 and Figure S3). Interestingly, we found better correlations when comparing the expression patterns of SynGAP isoforms with both synaptic markers than when comparing them with neuronal markers (Table 1). Indicating that, overall, the developmental expression of SynGAP isoforms parallels the formation of excitatory and inhibitory synapses rather than the differentiation of excitatory and inhibitory neurons. Indeed, only a few significant correlations where found between the expression levels of SynGAP isoforms and CaMK2α or GAD-67 (Table 1).

Nevertheless, we again observed a marked difference between the expression pattern of α1/α2 and β isoforms. While the former correlate very well with the expression of PSD-95 and Gephyrin in almost all tissues investigated, β isoforms present a more tissue-restricted correlation with synaptic markers (Table 1). Actually, β isoforms only present significant correlation with the expression of PSD-95 in hippocampus and striatum and with Gephyrin in hippocampus. Importantly, we observed very high levels of correlation between the developmental expression pattern of PSD-95 and Gephyrin in cortex, hippocampus, and striatum (Table 1), indicating that the incorporation of excitatory and inhibitory synapses occurs more or less simultaneously in these tissues (Oh, Lutzu, Castillo, & Kwon, 2016). Thus, it is not surprising that the expression of SynGAP isoforms correlates with PSD-95 but also with Gephyrin. Despite PSD-95 proteins may only interact directly with α1 isoforms (Kim et al., 1998), we also observed good expression correlation with PSD-95 and α2 isoforms (Table 1). Actually, the co-expression of PSD-95 with α1 and α2 isoforms presented a higher correlation with α2 isoforms, as these reached statistical significance in almost all brain areas investigated, while the correlation of PSD-95 and α1 isoforms was only significant in cortex and OB.

### 3.5 | SynGAP isoforms present different regional expression patterns

We next compared the abundance of SynGAP isoforms between brain regions in three postnatal developmental time points, PND4, 11, 21, and in young adults (PND56). At PND4 (Figure 4a), the expression of all isoforms investigated presented equivalent levels in cortex, hippocampus, and striatum, while cerebellar expression was always the lowest. In the OB, levels of α2 and β isoforms were un-distinguishable from those in the other forebrain areas, but α1 abundance was significantly reduced, presenting the same levels found in cerebellum. Actually, α1 isoforms present similarly low expression levels in cerebellum and OB in all ages investigated. At PND11, α2 and β-containing isoforms still present higher levels in OB when compared with cerebellum, yet this difference disappears at PND21 and 56, where both tissues express equally low levels of all isoforms.

After PND4, hippocampus was the region where SynGAP isoforms presented the highest levels (Figure 4b–d). This was already noticeable at PND11, although not all comparisons reached statistical significance, and very clear at PND56. Nevertheless, at PND21 cortical expression of α1 and α2 isoforms becomes more prominent. This phenomenon is particularly noticeable for the cortical expression of α1 isoforms, which present a cortex to hippocampus expression ratio close to 2 at PND21, and around 0.7 at PND11 and PND56. Instead the cortical increase of α2 isoforms at PND21 only brings them to the same levels found in hippocampus. This sharp increase in α1 abundance specific to cortical samples from PND21 animals exemplifies the specific regulation at which the expression of SynGAP isoforms can be.

### 3.6 | All SynGAP isoforms investigated are expressed in the young and old human cortex

Immunoblots of total cortical extracts from two human individuals with 19 and 67 years of age were performed to elucidate if SynGAP isoforms are expressed in this human tissue. We found expression of tSynGAP and all C-terminal variants in both samples (Figure S4). In line with the lower neuronal density found in the human cortex, as compared with the mouse one (Defelipe, Alonso-Nanclares, & Arellano, 2002), human immunoblots present a clear reduction per unit of protein of tSynGAP and all its isoforms. Interestingly, human samples presented the same two bands found in mice when investigated SynGAP isoforms by the same technique.

### 3.7 | Differential subcellular distribution of tSynGAP along cortical postnatal development and in adult hippocampus

Next, we investigated to what extent tSynGAP presented a differential subcellular distribution throughout postnatal development. This study was performed with mouse cortical samples. Five subcellular fractions were prepared: homogenate without nuclei (S1), cytosol, non-synaptic membranes (NSM), SNP and PSD (Figure S5). This fractionation protocol was applied to three postnatal development stages PND7, 14 and 21 and young adult (PND56). Protein yield (i.e., protein amount/ tissue weight) was calculated for all subcellular fractions generated (Figure S5a) and used, together with immunoblot intensity data (Figure S5b), to obtain a measure of protein abundance in each fraction. Interestingly, we observed a decrease in the SNP yield between PND14 and 21, simultaneous to an increase in the PSD yield, likely reflecting the increased maturation of synapses during this week (Figure S5c). Subcellular protein markers (GAPDH, Synaptophysin, Gephyrin and PSD-95) were used to validate the fractionation protocol (Figure S5d).

**FIGURE 5:**
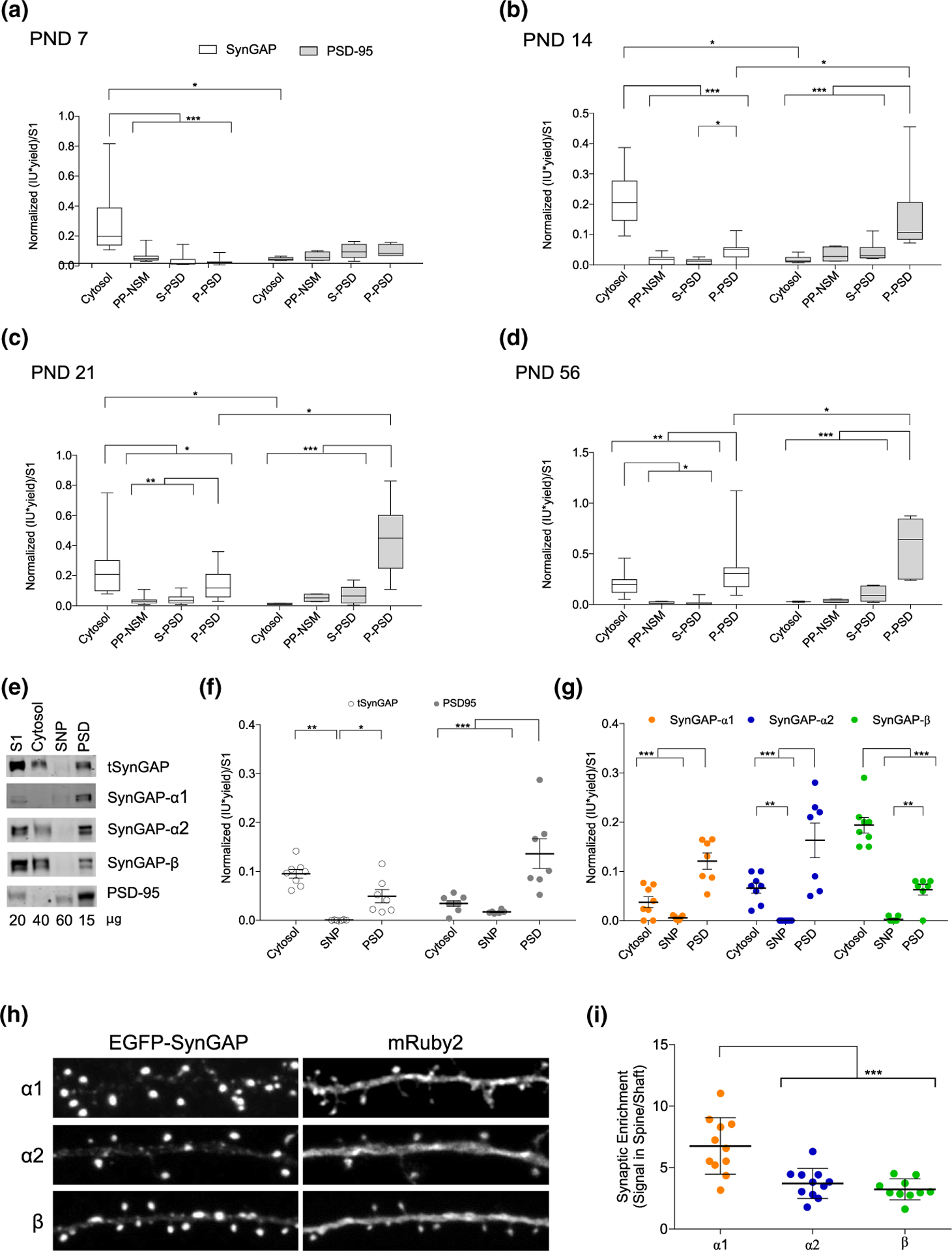
Subcellular distribution of total SynGAP (tSynGAP) and PSD-95 in cortex from four life stages and adult hippocampus. (a–d) box and whiskers plots representing the mean of normalized immunoblot intensity data (see Figure S5b) from different subcellular fractions. White boxes present tSynGAP data and gray boxes PSD-95 data. (*N*: postnatal day [PND]7 6–18, PND14 6–21, PND21 9–18, PND56 6–15). *N* indicates total number of technical replicates performed using cortical samples coming from six different mice. The standard error of the mean (*SEM*) is also shown. Mean differences were analyzed by two-way ANOVA followed by Fisher’s LSD post-hoc test, ***p < .001, **p < .01, and *p < .05. Subcellular fractions correspond with: cytosol; NSM, non-synaptic membranes; SNP, synaptic non-PSD and PSD, postsynaptic density. Life stages investigated are: PND7 (a), PND14 (b), PND 21 (c), and PND56 (d). (e) Immunoblots presenting subcellular distribution of total SynGAP (tSynGAP), its isoforms, and PSD-95 in adult (PND56) hippocampus. Subcellular fractions investigated: Homogenate without the nuclear fraction (S1); cytosol; SNP, synaptic non-PSD and PSD, postsynaptic density. (f) Dot plot with the mean of normalized immunoblot intensity data of tSynGAP (white dots) and PSD-95 (grey dots) in the subcellular fractions obtained from adult hippocampus (*N*: PND63 6–9 technical replicates using six biological replicates resulting from a pool of two mouse hippocampus per replica). (g) Dot plot with mean of normalized immunoblot intensity data of SynGAP isoforms containing α1, α2, and β C-terminal variants in the subcellular fractions obtained from adult hippocampus. *N*: PND63 6–8 technical replicates using six biological replicates resulting from a pool of the hippocampus from two mice per replica. The standard error of the mean (*SEM*) is also shown. Mean differences were analyzed by one-way ANOVA followed by Tukey’s post-hoc test ***p < .001, **p < .01, and *p < .05. (h) Mouse *Syngap1* KO hippocampal neurons (DIV17) transfected with constructs expressing enhanced green fluorescent protein (EGFP) tagged SynGAP C-terminal isoforms (α1, α2, and β) along with mRuby2 (cell-fill). (i) Synaptic enrichment is calculated as the ratio of EGFP signal present in dendritic spines versus dendritic shaft. *n* = 30 spines form 9–11 neurons for each isoform analysis. Error bars indicate *SEM*. Mean differences were assessed by one-way ANOVA followed by Tukey’s multiple comparison test, ***p < .0001

**FIGURE 6:**
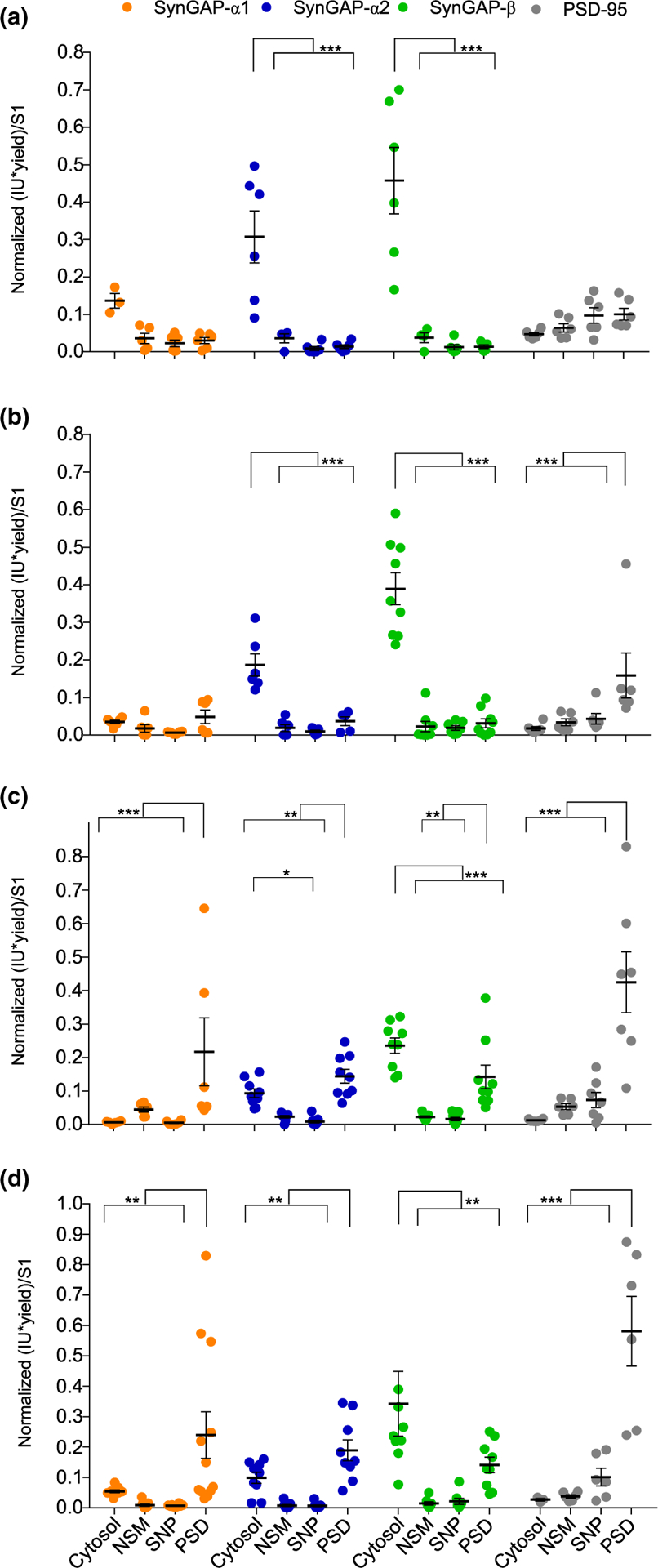
Subcellular distribution of SynGAP isoforms along three postnatal developmental stages and adulthood. (a–d) Dot plots representing the mean of normalized immunoblot intensity data (see Figure S5b) from different subcellular fractions for SynGAP isoforms presenting three different C-terminal variants and PSD-95. (*N*: PND7 6, PND14 6–9, PND21 6–9, PND56 6–11). *N* indicates total number of technical replicates performed using six different mouse cortical samples. The standard error of the mean (*SEM*) is also shown. Mean differences were analyzed by one-way ANOVA followed by Tukey’s post-hoc test, ***p < .001, **p < .01, and *p < .05. Subcellular fractions correspond with: cytosol; NSM, non-synaptic membranes; PND, postnatal day; SNP, synaptic non-PSD and PSD, postsynaptic density. Life stages investigated are: PND7 (a), PND14 (b), PND21 (c), and PND56 (d)

The abundance of tSynGAP in different subcellular fractions was analyzed by immunoblot together with that of PSD-95 (Figures 5–6). Of the four subcellular fractions investigated, tSynGAP was essentially found in two (Figure 5a–f), cytosol and PSD. For this reason we generated a PSD:cytosol expression ratio to analyze this data. The absence or minute amount of tSynGAP in NSM and SNP fractions indicated that it is not associated to extra-synaptic membranes and, furthermore, that within the synaptosome it is essentially found at the PSD. Interestingly, at PND7 tSynGAP was largely found in the cytosol (PSD:cytosol ratio 0.1) and it is not until PND56 that we observed significantly more tSynGAP in the PSD than in the cytosolic fraction from mouse cortices (Figure 5a–d). At PND14 and 21, the abundance of tSynGAP at the PSD was significantly higher than that found in the SNP fraction, yet tSynGAP was still more abundant in the cytosol. At PND21, the PSD:cytosol ratio of tSynGAP is still 0.6. Even at PND56, the remaining fraction of tSynGAP at the cytosol is quite large, as this ratio is 1.7. This developmentally regulated subcellular expression pattern of tSynGAP is in stark contrast with that displayed by PSD-95, as this scaffolding protein is predominantly expressed at the PSD in all life stages investigated, presenting almost negligible levels in the cytosolic fraction.

We also investigated adult (PND56) subcellular localization of tSynGAP and its isoforms in the hippocampus (Figure 5e–g). Here, we could not find a significantly different expression level of tSynGAP between cytosol and PSD, while PSD-95 presented a very restricted PSD localization. When considering the adult hippocampal expression of SynGAP isoforms we observed that α1 and α2 isoforms were enriched at the PSD, while β isoforms were very much enriched in the cytosolic fraction (Figure 5g). This is clearly indicated by the PSD:cytosol ratios found for these proteins: 3.2 for α1, 2.5 for α2, and 0.3 for β isoforms. We next aimed to validate the different subcellular localization of SynGAP isoforms observed in adult hippocampus using an alternative experimental approach. In this case we transfected primary cultures of hippocampal *Syngap1*^−/−^ neurons with GFP-tagged forms of SynGAP presenting one of the three C-terminal variants, finally, we quantified GFP signal in dendritic spines and shafts to obtain a ratio of spine:shaft GFP signal (Figure 5h,i). Notably, we did found a significant difference in the localization of α1 and the other two isoforms, being the later more prominently expressed at dendritic spines, in accordance with the immunoblot data collected from both cortical and hippocampal samples.

### 3.8 | Differential subcellular distribution of SynGAP isoforms along cortical postnatal development

We also investigated the subcellular distribution of SynGAP isoforms along cortical postnatal development (Figure 6 & Figure S5b). As expected, in this study we also localize all isoforms at two main subcellular locations, the cytosol and the PSD. We found very low levels of α1 isoforms early in postnatal development (PND7 & 14), as a consequence their subcellular location could not be confidently established at these ages (Figure 6a,b). At later ages, α1 isoforms presented a very restricted localization at the PSD (Figure 6c,d, PSD:cytosol ratio at PND21 and PND56 >4). Alpha2- and β-containing isoforms were expressed at higher levels early in development and could thus be localized at specific subcellular locations from PND7 to 56. These isoforms presented an almost exclusive cytosolic location during the first two postnatal weeks (Figure 6a,b, PSD:cytosol <0.2 in all cases), and a much-increased PSD localization between PND21 and PND56. Nevertheless, β isoforms were always found significantly more expressed in the cytosol than in the PSD, even at PND56 where the PSD:cytosol ratio is 0.4. In contrast, α2 isoforms present similar expression level in cytosol and PSD at both PND21 and 56, as indicated by the PSD:cytosol ratio, which is of 1.5 at PND21 and 1.9 at PND56, although PSD levels were found significantly higher. Therefore, subcellular localization of SynGAP isoforms in adult hippocampus recapitulate the findings found in adult cortex.

## 4 | DISCUSSION

It is established that mammals express multiple protein isoforms from the *Syngap1* gene, yet the complete set of SynGAP isoforms is still to be defined. Evidence from multiple sources, including transcriptomics data, suggest that human, mouse, and rat SynGAP isoforms would at least have 5 N-termini (A1, A2, B, C, and D) and 4 C-termini (α1, α2, β, and γ). Nevertheless, C and D N-termini have not been reported in humans and NCBI adds four extra N-termini (A3, A4, E, and F), and another C-terminus (α3), to the mouse set of variants. To the best of our knowledge, only the D N-terminus has been unambiguously identified at the protein level, which we report here for the first time. The fact that MS-based methods have not been able to identify unique peptides from the other isoforms, beyond peptides common to A1 and A2 (McMahon et al., 2012), suggests that their N-termini could be proteolyzed.

Different SynGAP isoforms have shown opposed effects in the control of synaptic strength (McMahon et al., 2012), to localize to different subcellular compartments (Li et al., 2001; Moon, Sakagami, Nakayama, & Suzuki, 2008; Tomoda, 2004) or to be able to bind to different proteins (Kim et al., 1998; Li et al., 2001), participating in different protein complexes and, by extension, in different molecular functions. SynGAP-β has even been described at the nucleus of cortical and hippocampal neurons (Moon et al., 2008), which would be in agreement with the signal that the Database of Nuclear Localization Signals (Bernhofer et al., 2017) identifies in the SynGAP core region (KKRKKD). This motif would be located toward the end of the C2 domain, as it occurs in the Doc2g protein, which is known to translocate to the nucleus through this signal (Fukuda, Saegusa, Kanno, & Mikoshiba, 2001). For this reason, it is essential to find out where and when these isoforms are expressed to have a correct understanding of SynGAP biology. Using three highly specific antibodies /against α1, α2, and β C-termini we compared isoform expression levels in different mouse brain regions along postnatal development and in adult, as well as their cortical and hippocampal subcellular distribution. We have also shown that isoforms presenting these three C-termini are also expressed in the cortex of two human individuals aged 19 and 67. As previously reported (Kim et al., 1998; Li et al., 2001; McMahon et al., 2012; Yang et al., 2013), the three antibodies identify two major bands in all conditions investigated, including in human samples. This indicates that each C-terminus will at least be expressed with two different N-termini. Importantly, we did not observe significantly different expression patterns between the top and bottom bands, indicating that the C-termini would determine the differential expression observed between isoforms.

Developmental expression in each of the five tissues analyzed revealed differences between SynGAP isoforms, although, overall, α1 and α2 isoforms presented a more similar expression pattern between each other than with β isoforms. Developmental expression differences were most noticeable in cortex, hippocampus, and striatum. As in cerebellum and olfactory bulb PND4 levels remained unaltered, for β isoforms, or just increased moderately, for α1 and α2 isoforms. In cortex and hippocampus, the temporal expression of α1 isoforms is importantly delayed relative to that of α2 and β, as α1 isoforms reach their maximum at PND56, while α2 and β at PND21. Thus, α1 isoforms would present very low levels at PND11, during the critical period of *Syngap1* deficiency (Clement et al., 2012, 2013), suggesting that other SynGAP isoforms may play a more relevant role early in this period. This is in agreement with our identification of unique ion peptide precursors through discovery MS studies for α2 and β from PND0 until PND56 and for α1 only in adulthood as well as with the observation that the PDZ binding motif found in α1 isoforms is not key for mediating SynGAP function in barrel cortex formation (Barnett et al., 2006). Interestingly, the developmental expression observed in striatum presents a bi-phasic pattern, which is unique to this brain region. Protein expression increases between PND4 and PND11, remains constant during the critical period, between PND11 and 21, and increases again between PND21 and PND56. This pattern is very obvious for α1 and α2 isoforms, while the second stage of increase, between PND21 and PND56, does not reach statistical significance for β isoforms.

Although SynGAP is a well-known component of excitatory synapses from principal neurons, it is also expressed in inhibitory neurons (Berryer et al., 2016) and it has been found co-localizing with Gephyrin and GAD-67 in primary neuronal cultures, especially β isoforms (Moon et al., 2008). We thus wanted to investigate if we could correlate the developmental expression pattern of SynGAP isoforms with the expression of markers of excitatory and inhibitory synapses and neurons, to investigate if different isoforms presented specific expression patterns relating them to the developmental processes of neurogenesis or synaptogenesis. Interestingly, we found that α1 and α2 isoforms better followed the expression of synaptic markers than neuronal ones, while β isoforms did not systematically correlate with neither synaptic nor neuronal markers. Noticeably, β isoforms only present significant expression correlation with Gephyrin in hippocampus, which is in agreement for a role of these isoforms in hippocampal inhibitory synapses, as previously reported (Moon et al., 2008).

Isoform abundance comparison between tissues revealed that cerebellum and olfactory bulb presented the lowest expression for all SynGAP isoforms, with the exception of α2 and β isoforms in the olfactory bulb at PND4. Isoform abundance comparison between cortex, hippocampus, and striatum revealed that at PND11 and adulthood the highest expression was found in the hippocampus. Yet, at PND21, toward the closure of the critical period, clear differences were observed between isoforms. At this stage, α1 isoforms presented the highest expression levels at cortex, α2 isoforms presented equivalent levels in cortex and hippocampus, while hippocampus remained the tissue with the highest expression of β isoforms. We therefore observed an increase in the cortical expression of α1 and α2 isoforms relative to their hippocampal levels, this being particularly striking for α1 isoforms. FMRP levels were found decreased specifically during PND21–23 in hippocampus from *Syngap1*^±^ mice to compensate the necessary increase in the expression of SynGAP mRNA at this point of development (Paul et al., 2019). Taken all these data together, PND21 is a key age for SynGAP neurobiology.

As SynGAP was first identified in the PSD, it has mainly been studied in the context of adult synaptic function. Yet, *Syngap1* expression starts early in embryogenesis (Porter, Komiyama, Vitalis, Kind, & Grant, 2005), before the onset of synaptogenesis, indicating that SynGAP must have non-synaptic functions. For this reason, we decided to investigate the distribution of SynGAP into subcellular compartments and if this changed along postnatal development. We looked for SynGAP distribution in four major cellular compartments, (a) cytosol, containing soluble proteins not bound to membranes such as GAPDH or Gephyrin, (b) non-synaptic membranes (NSM), (c) synapse excluding the PSD (SNP) which would include presynaptic proteins like Synaptophysin and (d) the PSD, and we compared it to the distribution of the PSD marker PSD-95. Although we detected SynGAP and its isoforms in all subcellular fractions investigated, when normalizing their abundance by the amount of protein found in each fraction, we observed that SynGAP largely partitions between two locations, the PSD and the cytosol. Indicating that SynGAP has a cytosolic function, which is essentially uncharacterized, and that this is the predominant function early in postnatal development.

tSynGAP subcellular distribution was remarkably different from that of PSD-95, which at PND7 was weakly expressed, but from PND14 onwards was almost exclusively found at the PSD. Instead, tSynGAP presented a clear cytosolic localization at all ages investigated, even in adult cortex and hippocampus. Actually, during the first two postnatal weeks, tSynGAP was almost exclusively found at the cytosol. The fraction of tSynGAP localized to the PSD, progressively increased along postnatal development but tSynGAP was only found significantly enriched in the PSD compared to cytosol in adult cortical samples. Even at PND21, when synaptogenesis is largely finished (Harris, 1999), tSynGAP was still found more abundant in the cytosol. It is also interesting to note that within synaptosomes, tSynGAP is almost exclusively found at the PSD, as we detected very low amounts of this protein at the SNP fraction, which contains all synaptic proteins that are not in the PSD. Different SynGAP isoforms presented a clearly distinctive subcellular localization pattern between the cytosol and the PSD. Being α1 and β isoforms the ones with the most opposed patterns. Alpha1 isoforms were always found highly restricted to the PSD, while β ones were always enriched in the cytosolic fraction and expressed with low levels at PSDs until PND21. Isoforms with the α2 C-termini presented an intermediate behavior, enriched in the cytosol in PND7 and 14 and enriched at the PSD at PND21 and 56. This differential distribution of SynGAP isoforms was also observed in hippocampal samples by two orthogonal methods, indicating that the same localization pattern could be found in other brain regions.

Interestingly, at PND14, when PSDs are already formed in the mouse cortex (Chandrasekaran et al., 2015; Swulius, Kubota, Forest, & Waxham, 2010), as indicated by the clear enrichment of PSD-95 in this fraction, α2 and β-containing isoforms present very low PSD levels. However, 1 week later (PND21), coinciding with the rise of α1 isoforms expression and localization to the PSD, the presence of α2 and β isoforms in this location clearly increases. Suggesting a cooperative mechanism of SynGAP isoforms localization to the PSD. An alternative explanation would be that between PND14 and PND21 the primary, and yet unknown, interacting point for SynGAP at the PSD becomes much more abundant, driving the increase in α2 and β isoforms at this location.

Taking into consideration that the interaction between SynGAP-α1 and PSD-95 is not required for SynGAP localization to the PSD (Barnett et al., 2006; Rumbaugh et al., 2006; Vazquez et al., 2004) we propose a model in which all SynGAP isoforms would be able to locate to the PSD through primary interaction(s) with unidentified protein(s). These putative interaction(s) would occur via a sequence within the core region of SynGAP. Furthermore, α1 isoforms would present a secondary PSD anchoring point at their PDZ binding motif, which would result in their increased stability at the PSD. This increased stabilization would not go in detriment of the well-described dispersion of α1 and α2 isoforms from the PSD upon synaptic activation, as dispersed isoforms rapidly return to the PSD (Araki, Zeng, Zhang, & Huganir, 2015; Yang et al., 2013; Yang, Tao-Cheng, Reese, & Dosemeci, 2011). Furthermore, the low abundance of α1 isoforms in the cytosol suggests that at any given time the proportion of α1 isoforms outside the PSD is relatively small.

In summary, we have identified clear developmental expression pattern differences between SynGAP isoforms, particularly during the critical period of *Syngap1* haploinsufficiency, which could have relevance to brain development and mental illness. Furthermore, we provide strong evidence showing that SynGAP, generally regarded as a protein exclusive to the PSD, is also found in the cytosol, where it is most abundant during postnatal brain development. Different isoforms present clearly distinctive subcellular distribution, being α1 isoforms highly restricted to the PSD, β ones mainly cytosolic and α2 isoforms presenting an intermediate behavior. We have observed that the presence of α2 and β isoforms in the PSD is developmentally regulated and coincides with the increased expression of α1 isoforms, while a non-synaptic role could be attribute to these less known isoforms. Understanding the functional differences between these isoforms will be key to disentangle the multiple functions performed by the SynGAP protein.

## Supplementary Material

supinfo1

## Figures and Tables

**TABLE 1:** Postnatal development expression correlation between SynGAP isoforms and synaptic and neuronal markers

	Pearson R correlation coefficient				
	Cortex	Hippocampus	Striatum	Olfactory bulb	Cerebellum
Expression correlation between SynGAP isoforms					
α1 versus α2	0.95* (0.048)	0.96* (0.040)	0.99* (0.007)	0.865	0.89
α1 versus β	0.75	0.84	0.88	0.40	−0.68
α2 versus β	0.90	0.96* (0.041)	0.93 (0.074)	0.36	−0.31
Expression correlation between PSD-95 and Gephyrin					
PSD-95 versus Gephyrin	0.98* (0.019)	0.99* (0.003)	0.99* (0.014)	0.12	0.90
Expression correlation between PSD-95 and SynGAP isoforms					
PSD-95 versus α1	0.98* (0.025)	0.95 (0.054)	0.94 (0.063)	0.98* (0.023)	0.89
PSD-95 versusα2	0.99* (0.005)	0.99* (0.004)	0.97* (0.030)	0.94 (0.056)	0.99* (4.7e5)
PSD-95 versus β	0.87	0.97* (0.029)	0.96* (0.043)	0.42	−0.31
Expression correlation between Gephyrin and SynGAP isoforms					
Gephyrin versus α1	0.93 (0.067)	0.97* (0.033)	0.93 (0.067)	0.18	0.61
Gephyrin versus α2	0.98* (0.023)	0.99* (0.003)	0.96* (0.042)	0.06	0.90
Gephyrin versus β	0.93 (0.067)	0.95* (0.049)	0.90	−0.81	0.13
Expression correlation between CaMK2α and SynGAP isoforms					
CaMK2α versus α1	0.78	0.93 (0.066)	0.49	0.76	−0.95* (0.049)
CaMK2α versus α2	0.89	0.99* (0.006)	0.59	0.97* (0.026)	−0.88
CaMK2α versus β	0.97* (0.03)	0.98* (0.021)	0.77	0.17	0.49
Expression correlation between GAD-67 and SynGAP isoforms					
GAD-67 versus α1	0.93 (0.075)	N/A	0.89	0.93 (0.067)	0.87
GAD-67 versus α2	0.99* (0.006)	N/A	0.94 (0.064)	0.97* (0.03)	0.95* (0.048)
GAD-67 versus β	0.90	N/A	0.97* (0.032)	0.74	−0.23

Abbreviations: N/A, not analyzed; PSD, postsynaptic density.

* Significant correlation, *p*-value between parenthesis (*p* ≤ 0.075 also included).

## Abbreviations:

C-term: C-terminal
DIV: days in vitro
DOC: sodium deoxycholate
EGFP: enhanced green fluorescent protein
FDR: false discovery rate
ID: intellectual disability
IP: immunoprecipitation
MS: mass spectometry
MS/MS: tandem mass spectrometry
N: number of technical replicas
N-term: N-terminal
ON: over-night
PBS: phosphate-buffered saline
PH: pleckstrin homology
PND: postnatal day
ppm: parts per million
PSD: postsynaptic density
PVDF: polyvinylidene fluoride
RRID: Research resource identifier
S1: homogenate without nuclei
SDS: sodium dodecyl sulfate
SNP: synaptic non-PSD
TBS: tris-buffered saline
tSynGAP: total SynGAP
ver.: version
α1: alpha1
α2: alpha2
β: beta
γ: gamma
