# Supplementary material for: SynGAP splice variants display heterogeneous spatio-temporal expression and subcellular distribution in the developing mammalian brain
Source: J Neurochem. Author manuscript; Available in PMC 2021 Sep 1. (PMC7754318; doi:10.1111/jnc.14988)
Supplement: supinfo1 [file NIHMS1572055-supplement-supinfo1.pdf]

SUPPLEMENTARY FIGURES AND TABLES

SynGAP Splice Variants Display Heterogeneous Spatio-Temporal Expression And Subcellular Distribution In The Developing Mammalian Brain

Gemma Gou, Adriana Roca-Fernandez, Murat Kilinc, Elena Serrano, Rita Reig-Viader, Yoichi Araki, Richard L. Huganir, Cristian de Quintana-Schmidt, Gavin Rumbaugh* and Àlex Bayés*

*** Corresponding authors**

Àlex Bayés, abayesp@santpau.cat

Molecular Physiology of the Synapse Laboratory

IIB Sant Pau

C/Sant Quintí, 77-79

08041 Barcelona, Spain

Gavin Rumbaugh, gavin@scripps.edu

Department of Neuroscience,

The Scripps Research Institute,

Jupiter, FL 33458, USA.

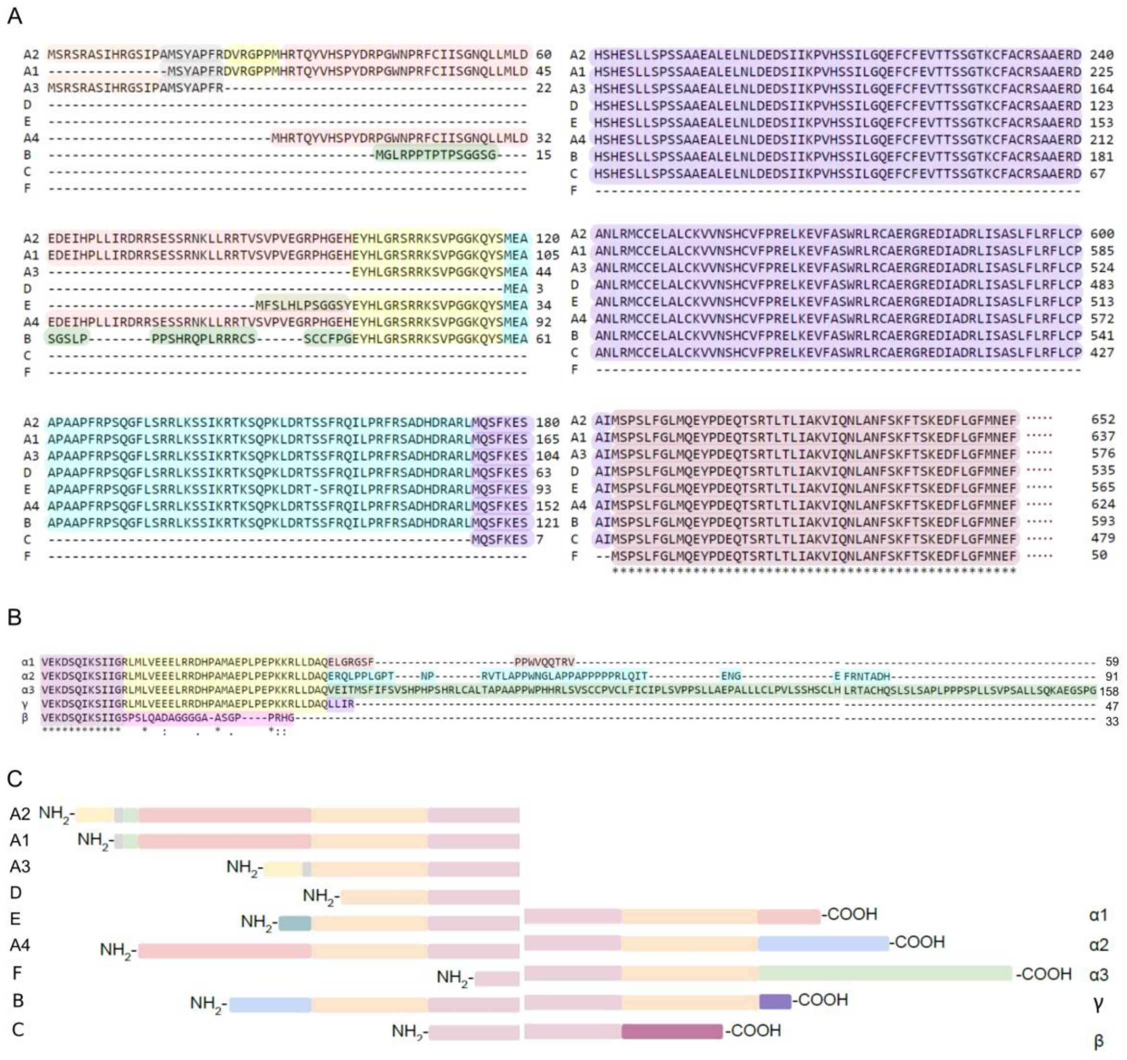

Supplementary Figure 1. SynGAP N- and C-terminal variants

A. Protein sequences from mouse SynGAP protein N-terminal variants.

B. Protein sequences from mouse SynGAP protein C-terminal variants.

C. Schematic representation of SynGAP protein N- and C-terminal variants. Fragments with the same color correspond with the same protein sequence. The beginning and the end of the SynGAP core region is also indicated. Note that this SynGAP core region is the sequence common to all isoforms without taking into account F-containing isoforms which lack a portion of it.

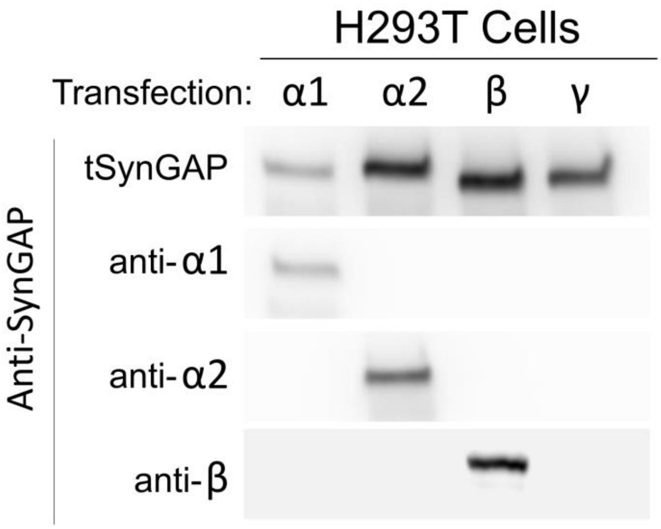

Supplementary Figure 2. Specificity of anti-SynGAP-β antibody.

Pools of HEK293T cells were transfected with GFP-tagged SynGAP cDNAs containing one of the four known C-terminal spliced sequences. Extracts from each cellular pool were immunoblotted with and antibody that was common to all SynGAP isoforms (tSynGAP) or antibodies raised against α1, α2, and β spliced variants.

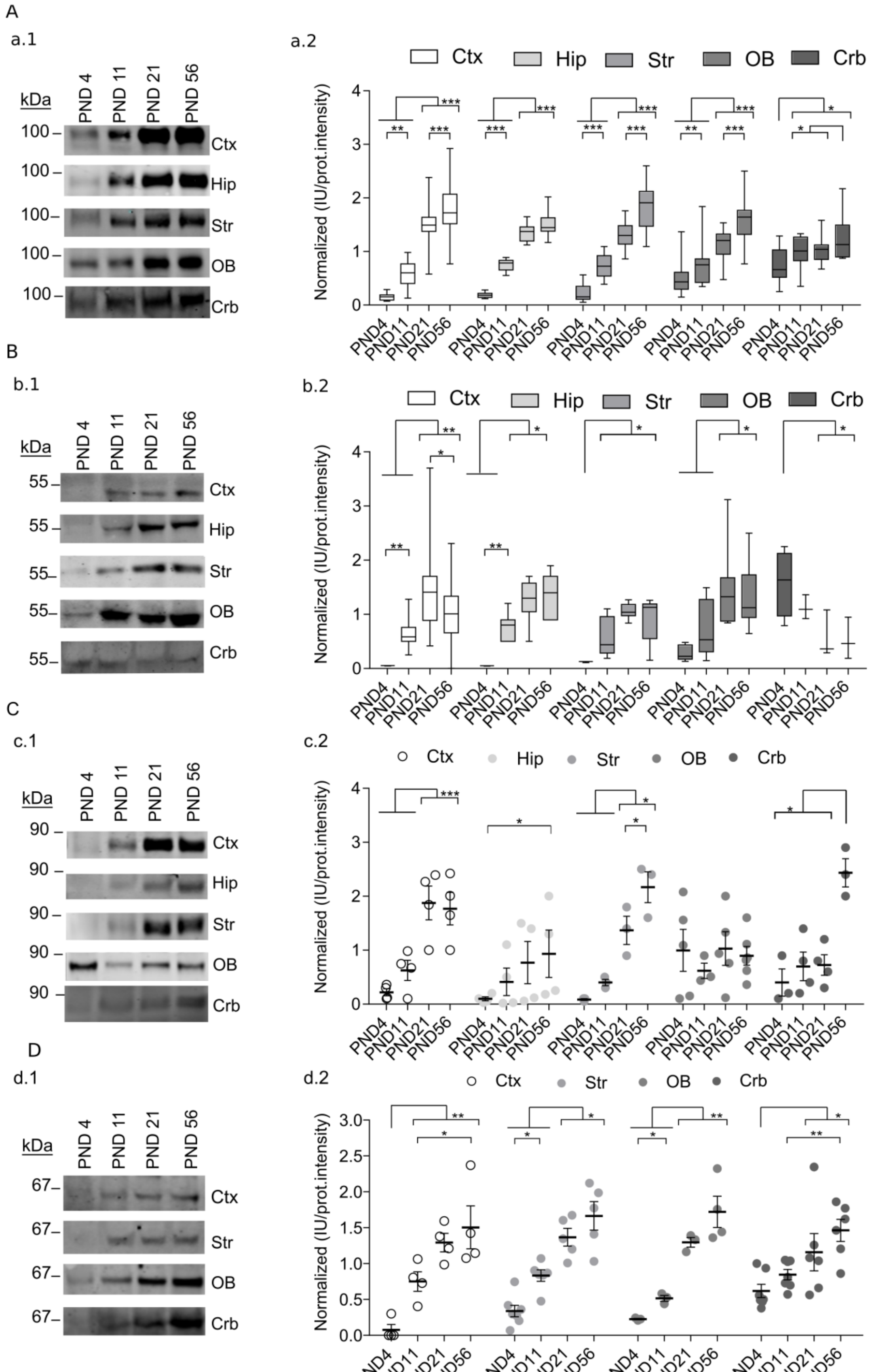
A
a.1
kDa
PND 4
PND 11
PND 21
PND 56
100
Ctx
Hip
Str
OB
Crb
a.2
Ctx
Hip
Str
OB
Crb
Normalized (IU/prot.intensity)
PND4
PND11
PND21
PND56
B
b.1
55
b.2
C
c.1
90
c.2
D
d.1
67
d.2

Supplementary Figure 3. Developmental Expression of synaptic and neuronal markers in five different brain regions.

A-D. Protein abundance in four life stages (PND4, 11, 21 and 56) and five brain regions: cortex (Ctx), hippocampus (Hip), striatum (Str), olfactory bulb (OB) and cerebellum (Crb) was investigated by immunoblot for PSD-95 (a1), CaMK2α (b1), Gephyrin (c1) and glutamate decarboxylase-1 (GAD-67, d1). **a2-d2** Box and whiskers or dot plots represent the mean of normalized immunoblot intensity data. N indicates total number of technical replicates from a pool of a given brain area coming from of a minimum of 2 mice (Fig. 1A). The standard error of the mean (SEM) is also shown. Mean differences were analyzed by one-way ANOVA followed by Tukey's post-hoc test, *** $p < 0.001$, ** $p < 0.01$ and * $p < 0.05$.

a.2. N: cortex 17-20, hippocampus 7-12, striatum 15-19, olfactory bulb 18 and cerebellum 11-13.

b.2. N: cortex 11-20, hippocampus 7-12, striatum 3-11, olfactory bulb 7-11 and cerebellum 3-4.

c2. N: cortex 4, hippocampus 3-4, striatum 3, olfactory bulb 3-5 and cerebellum 3-4.

d2. N: cortex 4, striatum 5, olfactory bulb 3-4 and cerebellum 6.

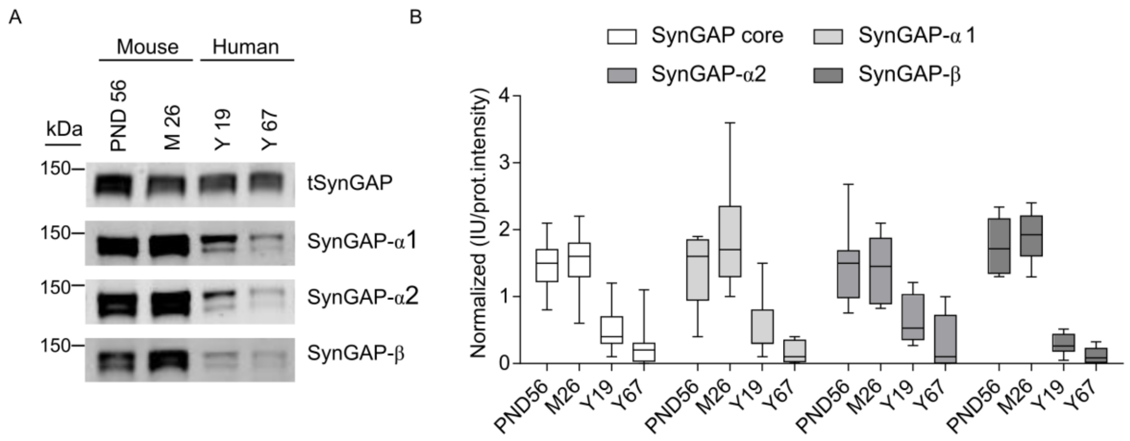

Supplementary Figure 4. Expression of SynGAP isoforms in human cortex.

A. Representative immunoblots showing expression of total tSynGAP as well as α1-, α2- and β-containing SynGAP isoforms in total protein extracts from human and mouse cortex. Two samples from human individuals aged 19 and 67 years were investigated. For comparison, mouse cortex homogenates at equivalent human ages (i.e. from PND56 and 26 months of age [M26] were analyzed.

B. Box and whiskers plots represent the mean of normalized immunoblot intensity data (N: mouse PND56 9-26, mouse 26 months (M26) 9-23, human 19 years 10-24, human 67 years 9-16). N indicates total number of technical replicates from a pool of cortical samples coming from two mice and one human per each age. The standard error of the mean (SEM) is also shown. No statistics were performed as we used one human sample per age.

A

Protein (µg)/weight (mg)				
	PND7	PND14	PND21	PND56
S1	56.1 +/-4.9	71.8 +/-8.3	71.4 +/-6.4	60.5 +/-4.6
Cytosol	31.5 +/-1.8	32.4 +/-2.6	34.4 +/-6	30 +/-1.7
NSM	2.4 +/-0.8	1.3 +/-0.5	3.3 +/-0.2	1.9 +/-0.2
SNP	10.2 +/-0.9	15 +/-2.3	10.2 +/-0.6	7.6 +/-0.4
PSD	1.6 +/-0.5	2.5 +/-0.8	6 +/-0.5	4.5 +/-0.4

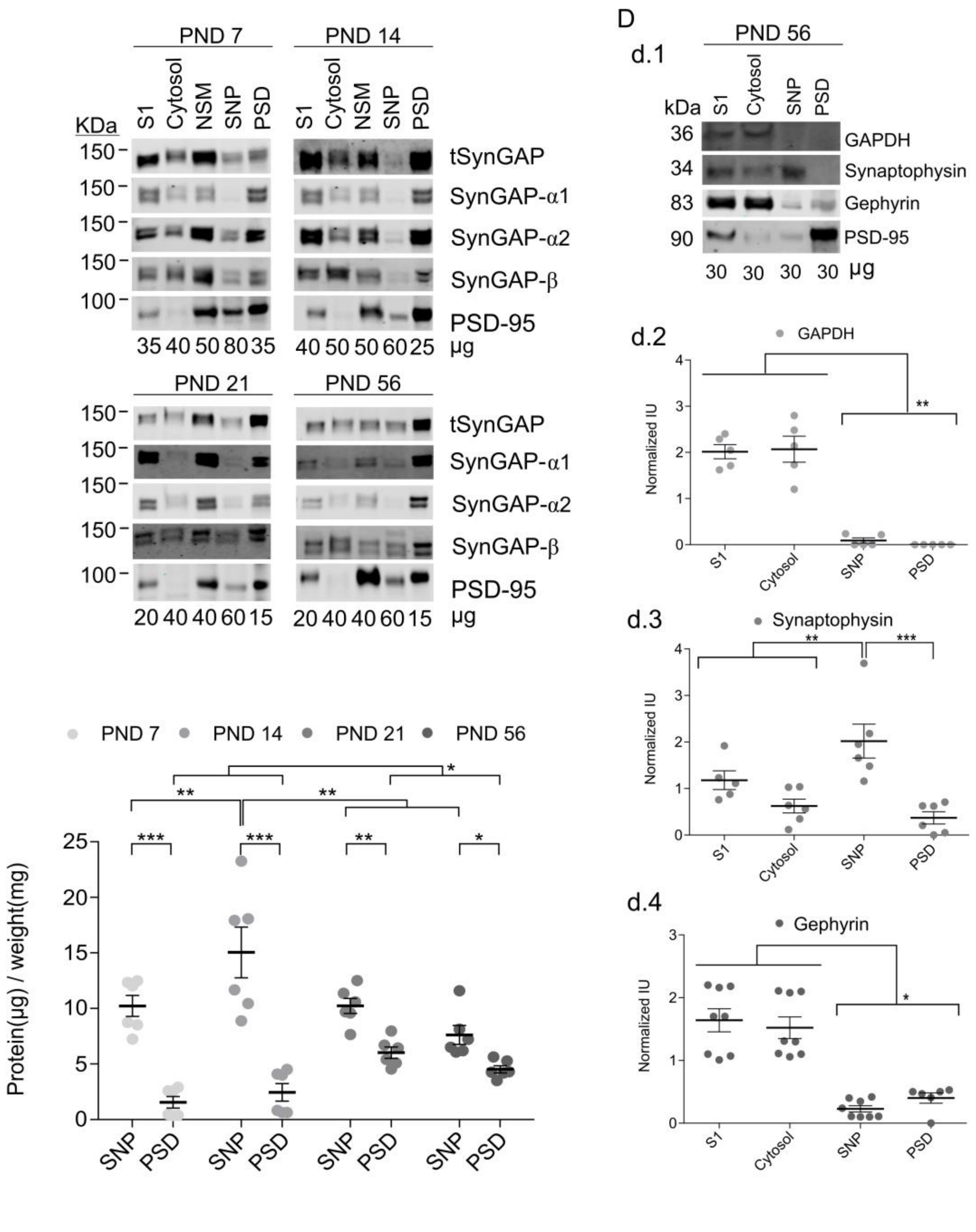

Supplementary Figure 5. Subcellular fractionation of mouse cortex and immunoblot analysis of SynGAP and its isoforms expression in four life stages.

A. Table presenting protein yields from all subcellular fractions generated. Protein yield is calculated as the ratio of total protein (in µg) in a given fraction by the weight (in mg) of the tissue used to obtain it. Subcellular fractions produced are: Homogenate without the nuclear fraction (S1); cytosol; NSM, non-synaptic membranes; SNP, synaptic non-PSD and PSD, postsynaptic density.

B. Representative immunoblots of tSynGAP, its isoforms and PSD-95 in the subcellular fractions produced are shown for each life stage analyzed. Protein amounts (µg) used for each fraction in immunoblots is indicated below each lane.

C. Protein yield of SNP and PSD fractions at the life stages investigated (PND7, 14, 21 and 56, N: 6 for all ages). The standard error of the mean (SEM) is also shown. The statistical test used was one-way ANOVA followed by Tukey's post-hoc test, *** $p < 0.001$, ** $p < 0.01$ and * $p < 0.05$.

D. Validation of the subcellular fractionation procedure by immunoblot (d1). Fractions investigated corresponded with homogenate without the nuclear fraction (S1), cytosol, synaptic non-PSD (SNP) and postsynaptic density (PSD). Markers of the cytosolic fraction (Glyceraldehyde-3-phosphate dehydrogenase, GAPDH, d2), the SNP fraction (Synaptophysin, d3) and PSD fraction (PSD-95) were used. The mainly cytosolic localization of the marker of inhibitory synapses (Gephyrin) is also shown (d4). **d.2.** N: 5 in all fractions. **d.3.** N: 6 in all fractions. **d.4.** N: 6-8 in all fractions. N indicates total number of technical replicates performed using two different mouse cortical samples.

MOUSE

ENSEMBL (Mouse)

Name	Transcript ID	Protein ID	Protein lenght	Estimated Molecular Weight (Da)	Biotype	CCDS	UniProt	N-term Variant	C-term Variant
Syngap1-203	ENSMUST00000193200.	ENSMUSP00000141245.2	1281	141,194.95	Nonsense mediated decay	-	A0A0A6YVS6	A1	Gamma
Syngap1-202	ENSMUST00000177932.	ENSMUSP00000137587.1	1308	144,866.22	Protein coding	-	J3QQ18	A2	Alpha1
Syngap1-213	ENSMUST00000229490.	ENSMUSP00000155085.1	1305	144,566.90	Protein coding	-	A0A2R8VHH2	A2	Alpha1
Syngap1-208	ENSMUST00000201702.	ENSMUSP00000144248.2	1289	142,828.87	Protein coding	-	A0A0J9YUM2	A2	Alpha1
Syngap1-204	ENSMUST00000194598.	ENSMUSP00000141686.1^	1340	148,238.03	Protein coding	CCDS59625	F6SEU4	A2	Alpha2
Syngap1-207	ENSMUST00000201349.	ENSMUSP00000144666.2[%]	1282	141,191.75	Protein coding	-	A0A0J9YVH8	A2	Beta
Syngap1-212	ENSMUST00000228963.	ENSMUSP00000154838.1	1246	137,203.54	Protein coding	-	A0A2R8VH83	B	Alpha1
Syngap1-201	ENSMUST00000081285.	ENSMUSP00000080038.4	1281	140,874.67	Protein coding	-	A0A140T8K9	B	Alpha2
Syngap1-214	ENSMUST00000231853.	ENSMUSP00000156340.1	1167	128,235.18	Protein coding	-	-	C	Alpha2

NCBI GENE (Mouse)

Name	Transcript ID	Protein ID	Protein lenght	Estimated Molecular Weight (Da)	N-term Variant	C-terminal Variant
Syngap1 isoform X6	XM_006524240.1	XP_006524303.1	1341	148,337.16	A2	Alpha2
Syngap1	NM_001281491.1	XP_920298^	1340	148,238.03	A2	Alpha2
Syngap1 isoform X1	XM_006524235.1	XP_006524298.1	1407	155,099.48	A2	Alpha 3*
Syngap1 isoform X2	XM_006524236.1	XP_006524299.1	1405	154,872.17	A2	Alpha 3*
Syngap1 isoform X3	XM_006524237.1	XP_006524300.1	1393	153,578.76	A2	Alpha 3*
Syngap1 isoform X11	XM_006524245.2	XP_006524308.1[%]	1282	141,191.75	A2	Beta
Syngap1 isoform X9	XM_006524243.1	XP_006524306.1	1296	143,522.80	A2	Gamma
Syngap1 isoform X7	XM_006524241.3	XP_006524304.1	1331	146,195.39	A3*	Alpha 3*
Syngap1 isoform X4	XM_006524238.3	XP_006524301.1	1379	152,016.93	A4*	Alpha 3*
Syngap1 isoform X5	XM_006524239.3	XP_006524302.1	1348	147,736.12	B	Alpha 3*
Syngap1 isoform X10	XM_006524244.3	XP_006524307.1	1290	141,547.08	D[&]	Alpha 3*
Syngap1 isoform X8	XM_006524242.3	XP_006524305.1	1321	145,011.99	E*	Alpha 3*
Syngap1 isoform X12	XM_006524248.1	XP_006524311.1	805	87,153.65	F*	Alpha 3*

#Possible sequencing error in first methionine. Protein could be expressed.
*Unpublished N- or C-terminal variants
^Equivalent proteins (same lenght, N- and C-term.)
[%]Equivalent proteins (same lenght, N- and C-term.)
[&]The D N-term from mouse and rat vary in their 3rd and 4th residues. Mouse presents MEAA and rat MEYF. We propose that the correct one for both species should be MEAA
CCDS: Consensus Coding Sequence

HUMAN

ENSEMBL (Human)

Name	Transcript ID	Protein ID	Protein lenght	Estimated Molecular Weight (Da)	Biotype	CCDS	UniProt	N-term Variant	C-term Variant
SYNGAP1-201	ENST00000293748.9	ENSP00000293748.6	1284	141,960.92	Nonsense mediated decay	-	A0A0A0MQZ2	A1	Gamma
SYNGAP1-228	ENST00000644458.1	ENSP00000495541.1	1308	144,612.87	Protein coding	-	A0A2R8Y6T2	A2	Alpha1
SYNGAP1-204	ENST00000449372.7	ENSP00000416519.4[$]	1292	142,864.84	Protein coding	-	B7ZCA0	A2	Alpha1
SYNGAP1-230	ENST00000646630.1	ENSP00000496007.1[%]	1343	148,284.00	Protein coding	CCDS34434	A0A1U9X8L0, Q96PV0	A2	Alpha2
SYNGAP1-202	ENST00000418600.7	ENSP00000403636.3	1285	141,247.77	Protein coding	-	Q96PV0	A2	Beta
SYNGAP1-207	ENST00000628646.2	ENSP00000486431.1	1299	143,568.78	Protein coding	-	Q96PV0	A2	Gamma
SYNGAP1-229	ENST00000645250.1	ENSP00000494861.1	1249	137,257.61	Protein coding	-	A0A2R8YDS2	B	Alpha1
SYNGAP1-203	ENST00000428982.4	ENSP00000412475.2	1284	140,928.74	Protein coding	-	Q96PV0	B	Alpha2

NCBI (Human)

Name	Transcript ID	Protein ID	Protein lenght	Estimated Molecular Weight (Da)	N-term Variant	C-term Variant
SYNGAP1 isoform 2	NM_001130066.1	NP_001123538.1[$]	1292	142,864.84	A2	Alpha1
SYNGAP1 isoform 1	NM_006772.2	NP_006763.2[%]	1343	148,284.00	A2	Alpha2

#Based on the protein sequence this transcript could be expressed
[%]Equivalent proteins (same lenght, N- and C-term.)
[$]Equivalent proteins (same lenght, N- and C-term.)
CCDS: Consensus Coding Sequence
(Cont.)

RAT

ENSEMBL (Rat)

Name	Transcript ID	Protein ID	Protein lenght	Estimated Molecular Weight (Da)	Biotype	CCDS	UniProt	N-term Variant	C-term Variant
Syngap1-201	ENSRNOT00000000572.	ENSRNOP00000000572.6[$]	1284	141,970.97	Protein coding		D3ZCL8	A1	Gamma
Syngap1-202	ENSRNOT00000040859.	ENSRNOP00000044041.3^	1308	144,622.92	Protein coding		F1LQW8	A2	Alpha1

NCBI (Rat)

Name	Transcript ID	Protein ID	Protein lenght	Estimated Molecular Weight (Da)	N-term Variant	C-term Variant
SYNGAP1 isoform 1	NM_181092.5	NP_851606.3^	1308	144,622.92	A2	Alpha1
SYNGAP1 isoform 2	NM_001113409.3	NP_001106880.2[%]	1249	137,259.56	B	Alpha1

Uniprot (Rat)

Uniprot-ID	Protein lenght	Estimated Molecular Weight (Da)	N-term Variant	C-term Variant
Q9QUH6-2/D3ZCL8[$]	1284	141,970.97	A1	Gamma
Q9QUH6-1/F1LQW8^	1308	144,622.92	A2	Alpha1
Q9QUH6-3[%]	1249	137,358.70	B	Alpha1
Q9QUH6-4	1171	128,489.47	C	Alpha2
Q9QUH6-5	1166	127,745.44	D[&]	Beta

Isoforms reported only in the literature (Rat)

Reference	Protein lenght	Estimated Molecular Weight (Da)	N-term Variant	C-term Variant
Chen et al. 1998	1293	143114.20	A1	Alpha1
Chen et al. 1998	1270	139749.09	A1	Beta
Chen et al. 1998	1226	133993.59	B	Beta
Kim et al. 1998	1135	124719.21	C	Alpha1

^Equivalent proteins (same lenght, N- and C-term.)

[%]Equivalent proteins (same lenght, N- and C-term.)

[&]The D N-term from mouse and rat vary in their 3rd and 4th residues. Mouse presents MEAA and rat MEYF. We propose that the correct one for both species should be MEAA

Supplementary Table 1. Mouse, rat and human *Syngap1*/*SYNGAP1* protein isoforms identified by Ensembl, NCBI or UniProt databases.

Variant	Unique Peptide Sequence	Ionic Precursor	Peak Area (Intensity Units)
		PND 0/1	
SynGAP-D	mEAAPAAPFRPSQGFLSR	995.9938++	1802994
SynGAP-D	mEAAPAAPFRPSQGFLSR	664.3316+++	1885129
SynGAP-α2	VTLAPPWNGLAPPAPPPPPR	1023.0702++	3585347
SynGAP-α2	VTLAPPWNGLAPPAPPPPPR	682.3825+++	8029659
SynGAP-α2	LQITENGEFR	603.8093++	25052336
SynGAP-α2	QLPPLGPTNPR	595.3380++	273971392
SynGAP-β	SIIGSPSLQADAGGGGAASGPPR	1012.0138++	42673360
SynGAP-β	SIIGSPSLQADAGGGGAASGPPR	675.0116+++	16715653
		PND 11	
SynGAP-D	mEAAPAAPFRPSQGFLSR	995.9938++	9702698
SynGAP-D	mEAAPAAPFRPSQGFLSR	664.3316+++	6188801
SynGAP-α2	QLPPLGPTNPR	595.3380++	138484432
SynGAP-α2	VTLAPPWNGLAPPAPPPPPR	1023.0702++	15871170
SynGAP-α2	VTLAPPWNGLAPPAPPPPPR	682.3825+++	28712010
SynGAP-α2	LQITENGEFR	603.8093++	26766820
SynGAP-β	SIIGSPSLQADAGGGGAASGPPR	1012.0138++	131803552
SynGAP-β	SIIGSPSLQADAGGGGAASGPPR	675.0116+++	52839004
		PND 21	
SynGAP-D	mEAAPAAPFRPSQGFLSR	995.9938++	16732052
SynGAP-D	mEAAPAAPFRPSQGFLSR	664.3316+++	11927298
SynGAP-α2	LQITENGEFR	603.8093++	36717168
SynGAP-α2	VTLAPPWNGLAPPAPPPPPR	1023.0702++	6693875
SynGAP-α2	VTLAPPWNGLAPPAPPPPPR	682.3825+++	12733106
SynGAP-α2	QLPPLGPTNPR	595.3380++	588498688
SynGAP-β	SIIGSPSLQADAGGGGAASGPPR	1012.0138++	136940752
SynGAP-β	SIIGSPSLQADAGGGGAASGPPR	675.0116+++	45366896
		PND 56	
SynGAP-D	mEAAPAAPFRPSQGFLSR	995.9938++	64078996
SynGAP-D	mEAAPAAPFRPSQGFLSR	664.3316+++	50273976
SynGAP-α1	GSFPPWVQQTR	651.8331++	11963907

SynGAP-α2	VTLAPPWNGLAPPAPPPPPR	1023.0702++	9078114
SynGAP-α2	VTLAPPWNGLAPPAPPPPPR	682.3825+++	15689616
SynGAP-α2	QLPPLGPTNPR	595.3380++	1868298624
SynGAP-β	SIIGSPSLQADAGGGGAASGPPR	1012.0138++	64078996
SynGAP-β	SIIGSPSLQADAGGGGAASGPPR	675.0116+++	50273976

Supplementary Table 2. N- and C-terminal variants from SynGAP identified by discovery MS from mouse cortex in four different postnatal ages.
